# The first mitochondrial genome of *Calophyllum soulattri* Burm.f.

**DOI:** 10.1038/s41598-024-55016-6

**Published:** 2024-03-01

**Authors:** Charles Anthon E. Cadorna, Dexter G. Pahayo, Jessica D. Rey

**Affiliations:** Plant Molecular Phylogenetics Laboratory, Institute of Biology, College of Science, University of the Philippines, Diliman, 1101 Quezon City, Philippines

**Keywords:** Cell biology, Genetics, Cell biology, Genetics

## Abstract

*Calophyllum soulattri* Burm.f. is traditionally used to treat skin infections and reduce rheumatic pain, yet genetic and genomic studies are still limited. Here, we present the first complete mitochondrial genome of *C. soulattri*. It is 378,262 bp long with 43.97% GC content, containing 55 genes (30 protein-coding, 5 rRNA, and 20 tRNA). Repeat analysis of the mitochondrial genome revealed 194 SSRs, mostly mononucleotides, and 266 pairs of dispersed repeats ($\ge$30 bp) that were predominantly palindromic. There were 23 homologous fragments found between the mitochondrial and plastome genomes. We also predicted 345 C-to-U RNA editing sites from 30 protein-coding genes (PCGs) of the *C. soulatrii* mitochondrial genome. These RNA editing events created the start codon of *nad1* and the stop codon of *ccmFc*. Most PCGs of the *C. soulattri* mitochondrial genome underwent negative selection, but *atp4* and *ccmB* experienced positive selection. Phylogenetic analyses showed *C. soulattri* is a sister taxon of *Garcinia mangostana*. This study has shed light on *C. soulattri’s* evolution and Malpighiales’ phylogeny. As the first complete mitochondrial genome in Calophyllaceae, it can be used as a reference genome for other medicinal plant species within the family for future genetic studies.

## Introduction

*Calophyllum soulattri* Burm.f. is a medicinally important evergreen tree belonging to the Calophyllaceae family of the order Malpighiales^1^. The natural distribution of this species extends from South Indo-China to the Caroline Islands (http://www.plantsoftheworldonline.org/). Its leaves, roots, and stem bark have been shown to exhibit antimicrobial, cytotoxic, and anti-obesity properties^2,3^. In *C. soulattri*, the most active secondary metabolites were calosubellinone, garsubellin B, and soulattrin^4,5^. Generally, the genus Calophyllum contains a diverse array of bioactive constituents, including xanthones and coumarins^3^.

Cellular respiration is a complex metabolic process involving the breakdown of sugar molecules in the presence of oxygen to generate adenosine triphosphate (ATP) which occurs in mitochondria^6,7^. Apart from their primary function in ATP production, mitochondria actively participate in several metabolic pathways. Notably, they are involved in the biosynthesis of specific amino acids, lipids, and heme–a crucial component of various enzymatic systems^8^. Moreover, mitochondria are thought to have originated from an ancient endosymbiotic event, wherein a free-living bacterium is engulfed by a eukaryotic cell^9^. This endosymbiotic theory accounts for the presence of a double membrane in mitochondria, along with their distinct circular DNA, resembling that of bacterial DNA^10^.

The plant mitochondrial genome typically consists of a circular DNA molecule, with sizes ranging from approximately 66 kb^11^ to about 11.7 Mb^12^ among the genomes sequenced to date. Nonetheless, subgenomic forms also exist because of repeat-mediated homologous recombination^13^. Conversely, plastomes have a more consistent regular structure characterized by a highly conserved arrangement of genes in a circular DNA molecule^14^. This is why the focus has predominantly been on plastomes in the realm of plant phylogenetic studies. In addition, the chloroplast genome of *C. soulattri* has been recently reported^15^. To date, the number of chloroplast genomes deposited in NCBI GenBank (https://www.ncbi.nlm.nih.gov/genome/browse#!/organelles/) is more than 9,000; along with more than 1,000 plastid genomes. In contrast, only more than 540 plant mitochondrial genomes are available in the database. However, no mitochondrial genome from the Calophyllaceae family has been included in the NCBI GenBank Organelle Genome repository. But several mitogenome studies have already been reported in the order Malpighiales such in *Salix* species, *Bruguiera sexangula*, *Passiflora edulis*, *Populus* species, and *Garcinia mangostana*^16–20^. The differences in the number of organelle genomes can be explained by the difficulties encountered during the assembly process. Mitochondrial genomes often possess repetitive regions and frequently undergo rearrangement events, which present challenges when traditional short-read sequencing methods are used for their assembly. Therefore, the process of obtaining complete and accurate mitochondrial genome sequences is more intricate than that of plastomes and often requires the application of advanced sequencing technologies^21^. Nevertheless, complete plant mitochondrial genomes have been successfully generated using traditional short reads^22–24^.

The number of protein-coding genes (PCGs) and introns in the mitochondrial genome are also highly diverse^11,25–27^. The angiosperm mitochondrial genome has two types of introns (Group I and Group II) that are distinguished based on their conserved folding structures and splicing mechanisms^28^. These introns undergo *cis*- or *trans*-splicing to generate continuous and functional transcripts^29^.

In this study, we successfully assembled the first complete mitochondrial genome of *C. soulattri*. This computationally feasible assembly, solely from Illumina short reads, enabled us to investigate and reveal genomic characteristics and structural features in detail. Our analysis examined the relative synonymous codon usage (RSCU) and repeat sequences. Additionally, we explored the intriguing possibility of chloroplast DNA transfer into the mitochondrial genome. We also compared the gene content of the mitochondrial genome with that of other closely related species of the order Malpighiales. Furthermore, we probed RNA editing sites and analyzed the selective pressure of PCGs. Finally, we studied the phylogenetic relationship of *C. soulattri* with other representative species from the order Malpighiales. The findings from this study provide valuable information regarding the evolutionary dynamics of *Calophyllum* species at the mitochondrial level. These results serve as a fundamental resource for the development of vital genetic tools for *C. soulattri*.

**Figure 1:**
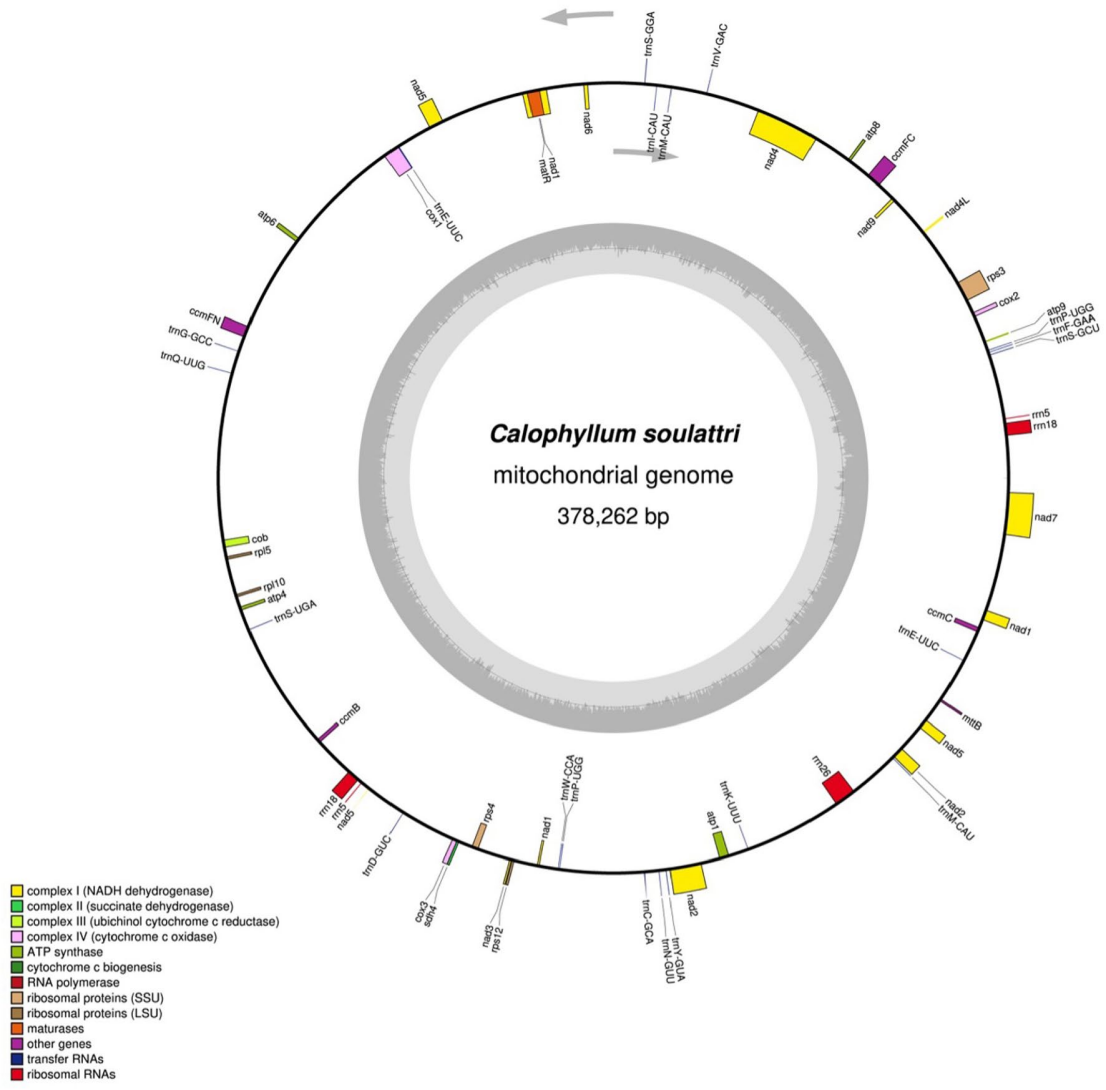
The circular map of *Calophyllum soulattri* mitochondrial genome. The gray arrows show the direction of transcription. Genes inside the circle are transcribed clockwise while genes outside the circle are transcribed counterclockwise. The gray bars inside the circle represent the GC content of the sequence.

**Table 1:** Annotation of genes in *Calophyllum soulattri* mitochondrial genome.

**Group**	**Genes**
Subunit of ATPase	*atp1*, *atp4*, *atp6*, *atp8*, *atp9*
Cytochrome c biogenesis	*ccmB*, *ccmC*, *ccmFc*, *ccmFn*
Apocytochrome b	*cob*
Subunit of cytochrome c oxidase	*cox1*, *cox2*, *cox3*
Maturase R	*matR*
Transport membrane protein	*mttB*
Subunit of NADH dehydrogenase	*nad1*, *nad2*, *nad3*, *nad4*, *nad4L*, *nad5*, *nad6*, *nad7*, *nad9*
Small subunit of ribosome	*rps12*, *rps3*, *rps4*
Large subunit of ribosome	*rpl10*, *rpl5*
Subunit of succinate dehydrogenase	*sdh4*
rRNA	*rrn18* ($\times 2$), *rrn26*, *rrn5* ($\times 2$)
tRNA	*trnE*-UUC ($\times 2$), *trnM*-CAU ($\times 2$), *trnP*-UGG ($\times 2$), *trnC*-GCA, *trnD*-GUC, *trnF*-GAA, *trnG*-GCC, *trnK*-UUU, *trnl*-CAU, *trnN*-GUU, *trnQ*-UUG, *trnS*-GCU, *trnS*-GGA, *trnS*-UGA, *trnV*-GAC, *trnW*-CCA, *trnY*-GUA

**Figure 2:**
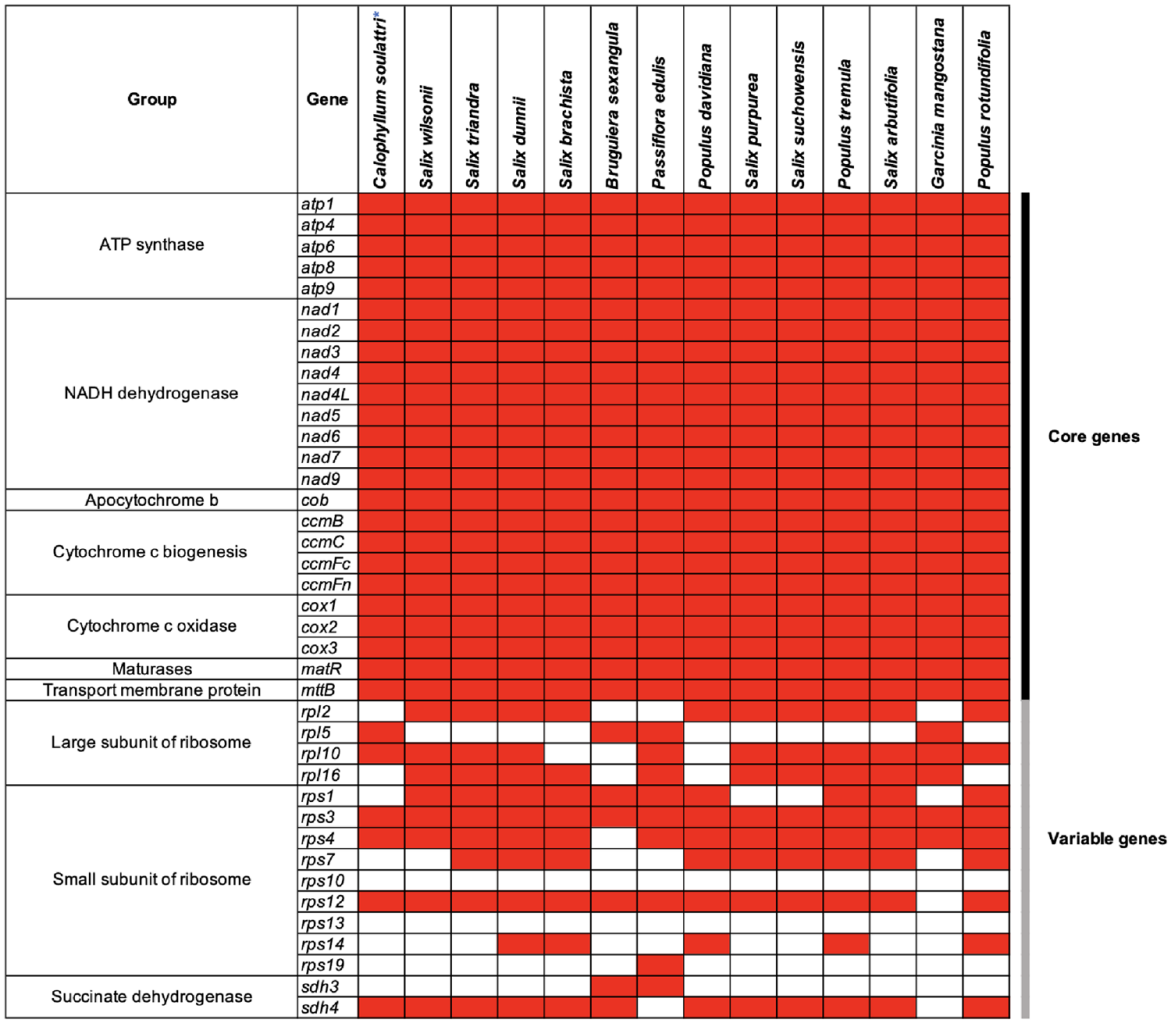
Distribution of protein-coding genes in the mitogenomes of representative species in the order Malpighiales. Red boxes indicate that the gene is present while white boxes indicate that the gene is absent. *Calophyllum soulattri* is labelled with an asterisk (*).

**Figure 3:**
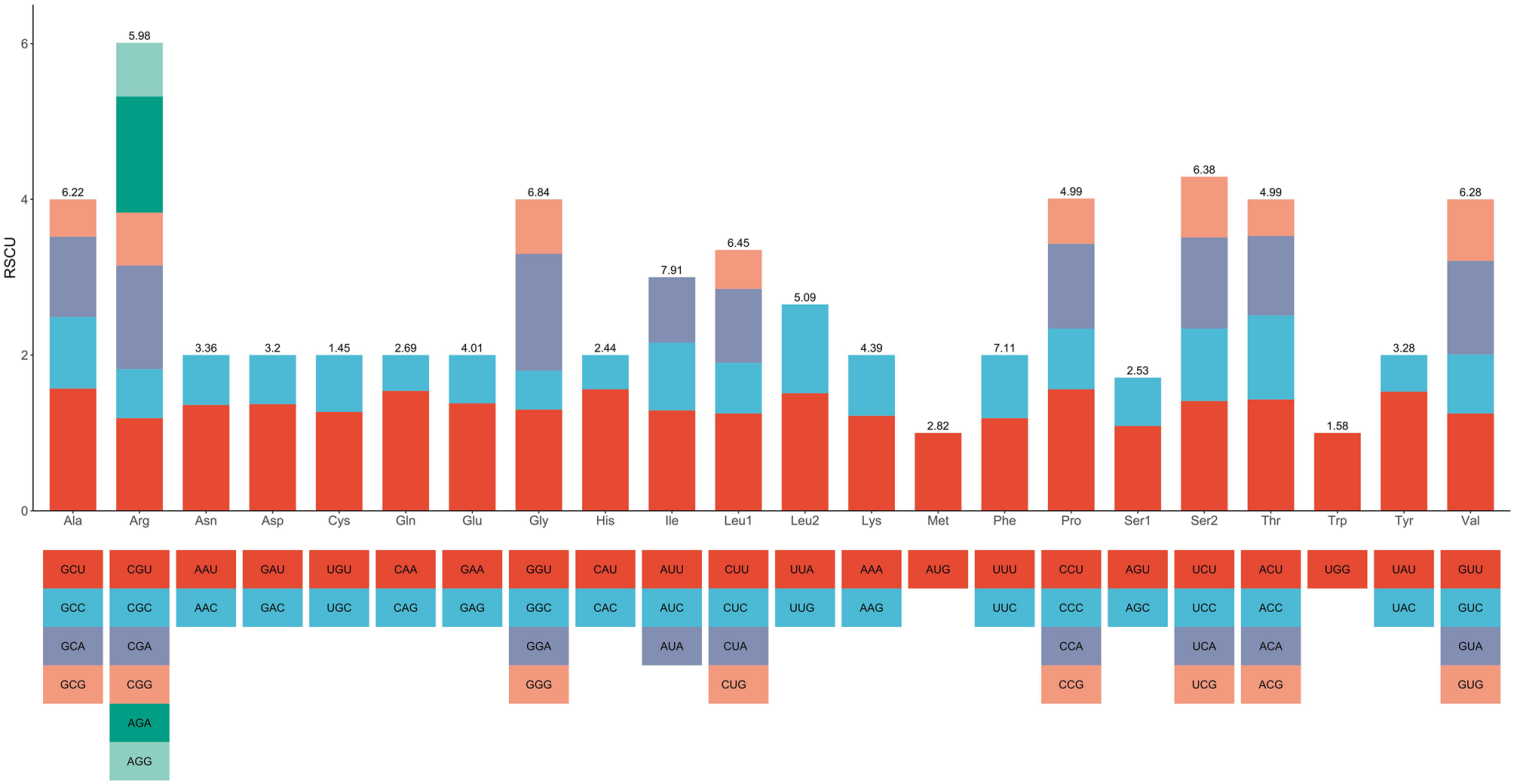
Codon usage bias of the mitochondrial PCGs of *Calophyllum soulattri*. The RSCU refers to relative synonymous codon usage. Codon families are labelled on the x-axis. Values on top of the bars refer to amino acid usage.

**Figure 4:**
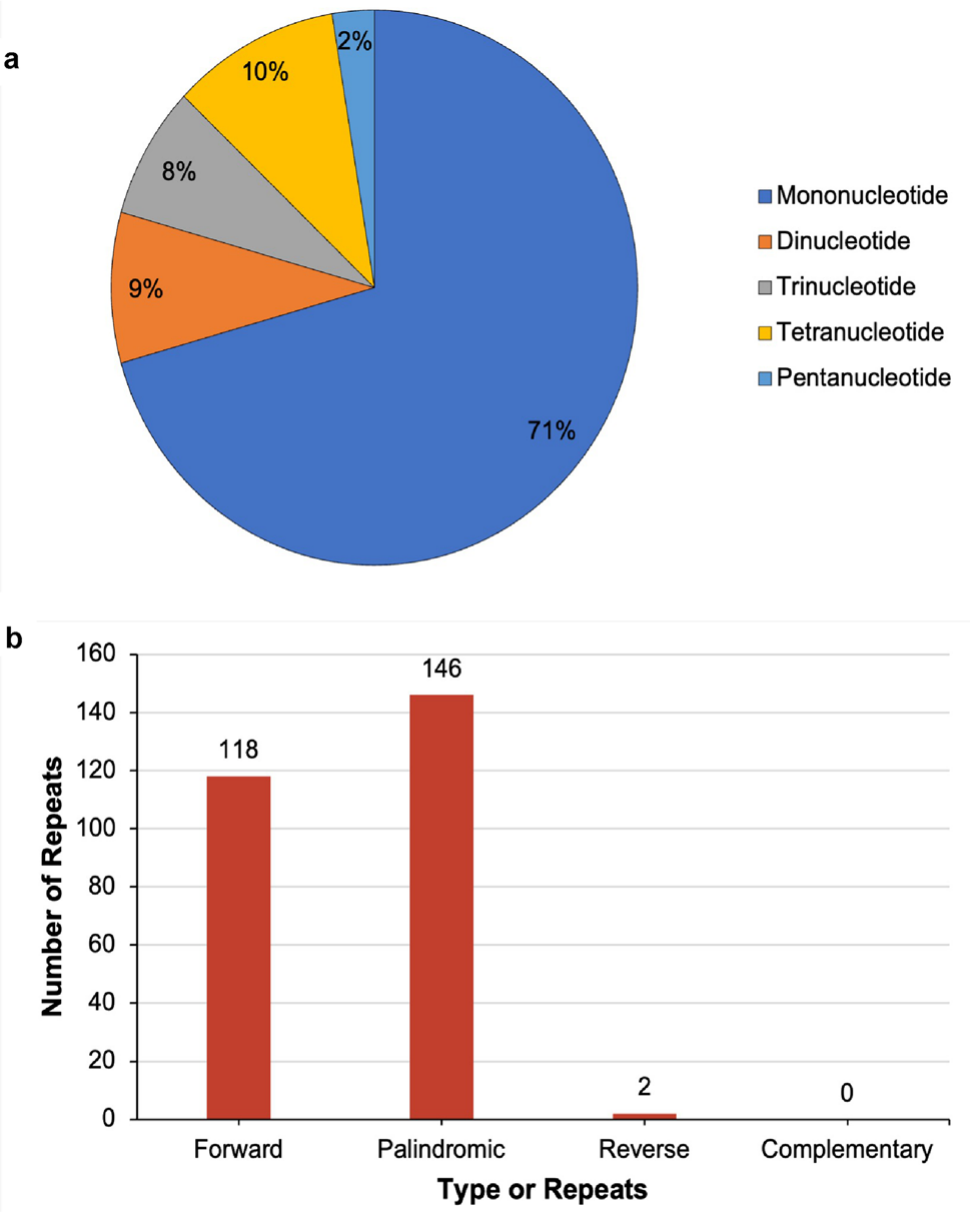
Repetitive elements in the mitochondrial genome of *Calophyllum soulattri*. (**a**) Abundance of simple sequence repeats (SSRs), and (**b**) the number and types of dispersed repeats ($\ge$30bp) identified in *C. soulattri* mitochondrial genome.

**Figure 5:**
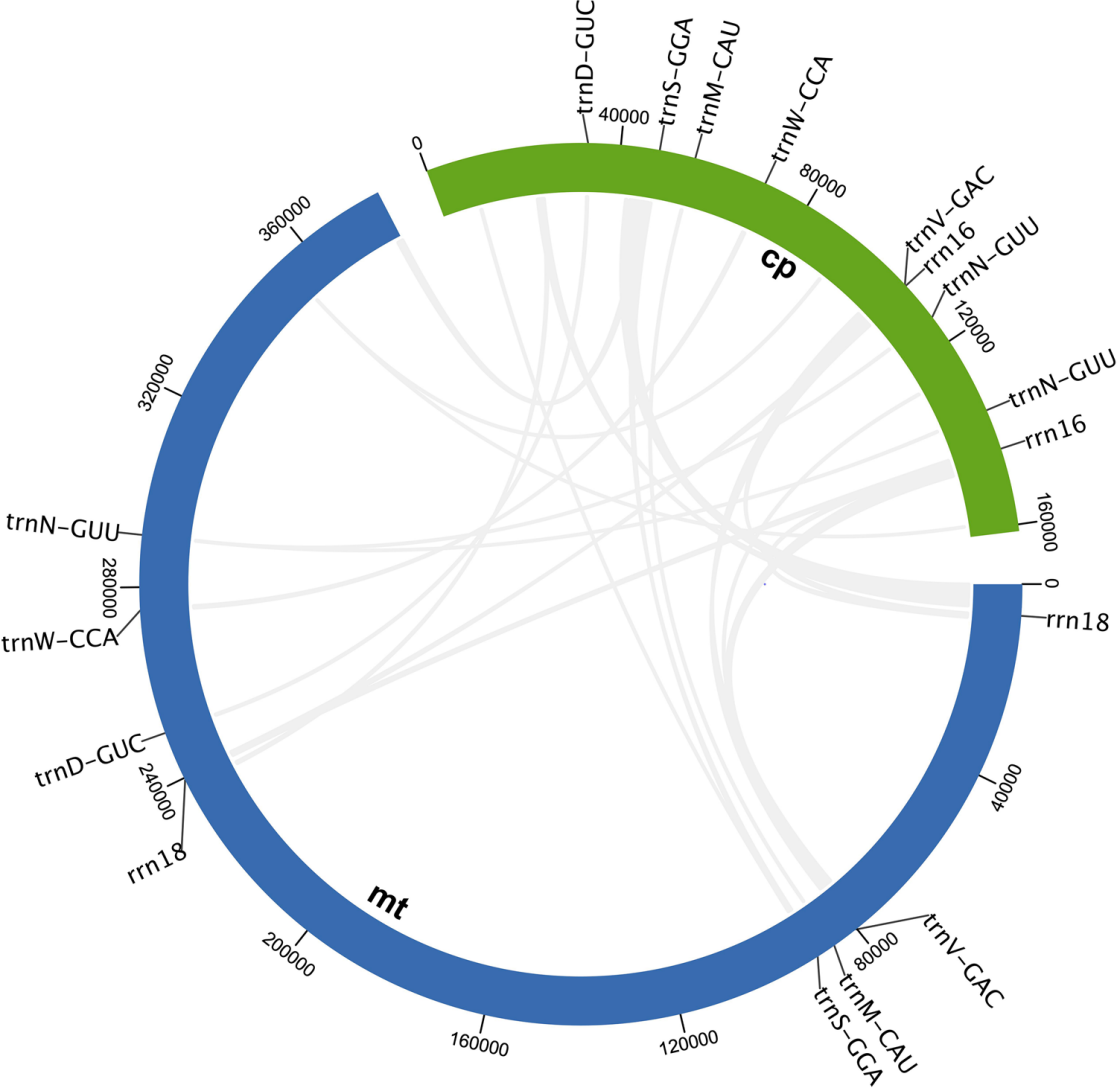
Circular map showing the distribution of MTPTs on the mitochondrial genome of *Calophyllum soulattri*. The blue and green outer arcs represent the mitochondrial genome (mt) and chloroplast genome (cp), respectively. The gray links in the inner circle represent the MTPTs on *C. soulattri* mitochondrial genome.

**Figure 6:**
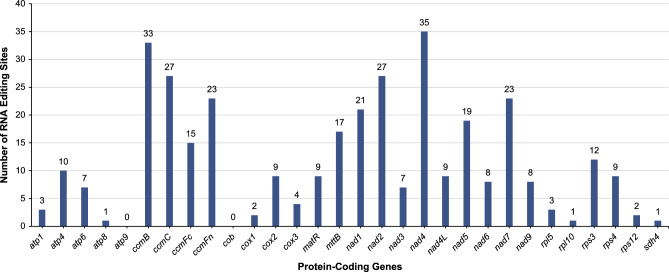
Number of RNA editing sites identified in the PCGs of *Calophyllum soulattri* mitochondrial genome. The abscissa shows the name of the genes, while the ordinate shows the number of edited sites.

**Figure 7:**
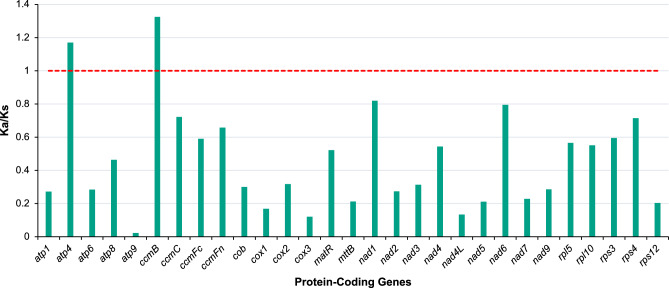
The Ka/Ks ratios for 29 protein-coding genes of *Calophyllum soulattri*. The red broken line represents Ka/Ks ratio of 1.0, indicating neutral selection.

**Figure 8:**
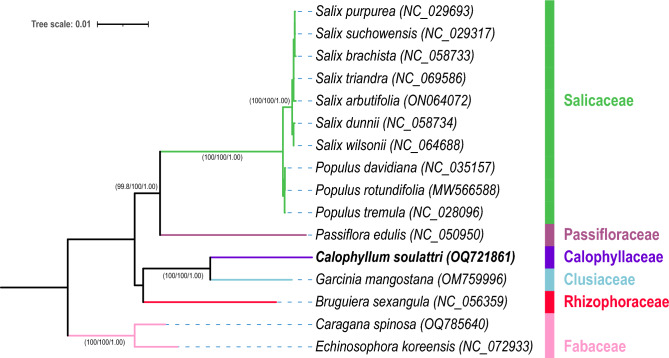
Phylogenetic tree of 14 plant species from the order Malpighiales and two non-Malpighiales species inferred from IQ-TREE and MrBayes based on 25 concatenated orthologous mitochondrial PCGs with the best-fit partitioning strategy. Two species from Fabaceae (*Caragana spinosa* and *Echinosophora koreensis*) were used as outgroups. Values inside parenthesis beside branches are SH-aLRT/UFBoot/PP support. The tree scale indicates the number of nucleotide substitutions per site. The mitochondrial genome obtained from this study was highlighted in bold.

## Results

### The general structure and gene composition of *C. soulattri* mitochondrial genome

The mitochondrial genome of *C. soulattri* is circular and 378,262 base pairs (bp) in length with a total GC content of 43.97% (Fig. 1). This is the first complete mitochondrial genome of the species and in the Calophyllaceae family. The average sequencing depth of the genome was $87.3\times$ (Supplementary Figure S1c). It also contained 55 annotated genes of which 50 were unique (Table 1) and included 30 protein-coding genes (PCGs). The annotated core PCGs in the mitochondrial genome of *C. soulattri* included five ATP synthase genes (*atp1*, *atp4*, *atp6*, *atp8*, and *atp9*), four cytochrome biogenesis genes (*ccmB*, *ccmC*, *ccmFc*, and *ccmFn*), three cytochrome oxidase genes (*cox1*, *cox2*, and *cox3*), nine NADH dehydrogenase genes (*nad1*, *nad2*, *nad3*, *nad4*, *nad4L*, *nad5*, *nad6*, *nad7*, and *nad9*), one apocytochrome b gene (*cob*), one maturase R gene (*matR*), and one membrane transport protein gene (*mttB*). The annotated variable PCGs in the mitochondrial genome of *C. soulattri* included the following: three small subunit of ribosomal proteins (*rps3*, *rps4*, and *rps12*), two large subunit of ribosomal proteins (*rpl5* and *rpl10*), and one succinate dehydrogenase gene (*sdh4*). Furthermore, the mitochondrial genome harbored 17 distinct tRNA genes and three distinct rRNA genes, which were annotated. Some tRNA genes had two copies such as *trnE*-UUC, *trnM*-CAU, and *trnP*-UGG. Similarly, there were rRNA genes with two copies, in particular *rrn18* and *rrn5*.

Among the 30 PCGs, nine had introns (Supplementary Figure S2). These included genes with a single *cis*-spliced intron, particularly *ccmFc*, *cox1*, *rps3*, and *trnE*-UUC. We also identified PCGs with many introns. Specifically, *nad4* contains three *cis*-spliced introns, whereas *nad5* contains two *trans*-spliced introns and two *cis*-spliced introns. On the other hand, *nad7* contains four *cis*-spliced introns. Moreover, *nad1* contains two *trans*-spliced introns and two *cis*-spliced introns while *nad2* harbored one *trans*-spliced intron and three *cis*-spliced introns.

### Comparison of gene content with other related Malpighiales mitochondrial genome

The gene content of *C. soulattri* was analyzed and compared with that of other species in the order Malpighiales (Fig. 2 and Supplementary Table S2). Our findings showed that all the core genes were present in every species. Additionally, it is evident that not all species contain the same set of large and small subunits of ribosome genes, as well as succinate dehydrogenase genes. Furthermore, the mitochondrial genome of *C. soulattri* was found to have lost nine variable genes, namely, *rpl2*, *rpl16*, *rps1*, *rps7*, *rps10*, *rps13*, *rps14*, *rps19*, and *sdh4*.

### Analysis of codon usage bias

Codon usage analysis was conducted on 30 PCGs within the mitochondrial genome of *C. soulattri* (Fig. 3 and Supplementary Table S4). Codons exhibiting a relative synonymous codon usage (RSCU) value greater than 1 were preferentially utilized by specific amino acids, revealing a prevalent universal codon usage pattern among mitochondrial PCGs. Interestingly, alanine (Ala) exhibited a pronounced preference for GCU codons, with the highest RSCU value observed among mitochondrial PCGs (RSCU = 1.57). This was followed by histidine (His) and proline (Pro), with a strong bias towards CAU and CCU codons, respectively (RSCU = 1.56). The codons UUU (Phe), AUU (Ile), and UUA (Leu) emerged as the most frequently used in *C. soulattri*. These observations suggest that codon preferences likely evolved through extensive selective pressures acting on *C. soulattri* over an extended evolutionary timeframe.

### Repeat elements of *C. soulattri* mitochondrial genome

Simple sequence repeats, also known as microsatellites or short tandem repeats (STR), are repetitive DNA sequences consisting of short units of nucleotides, typically 1-6 base pairs^30^. We identified 194 SSRs in *C. soulattri* mitochondrial genome. Most of these (137 repeat units) were mononucleotide repeats, accounting for 70.62% of the total SSRs (Fig. 4A). This was followed by tetranucleotides, which consisted of 20 repeat units (10.31%). In addition, we identified 17 dinucleotide repeat units, 15 trinucleotide repeat units, and 5 pentanucleotide repeat units, accounting for 8.76%, 7.73%, and 2.58% of the total SSRs, respectively.

Dispersed repeats were detected within the mitochondrial genome of *C. soulattri* (Fig. 4B). The majority of repeats were comprised of both palindromic and forward sequences. There were 266 pairs of repetitive sequences, each with a length equal to or exceeding 30 bp. Among these pairs, 146 pairs were identified as palindromic repeats, 118 as forward repeats, and two as reverse repeats. However, no instances of complementary repeats have been identified in the mitochondrial genome of *C. soulattri*.

Most dispersed repeats were shorter than 100 bp, with only nine repeats exceeding 100 bp. Notably, the longest forward repeat identified was 10,913 bp in length, followed by a forward repeat with a length of 1,140 bp (Supplementary Table S5). The combined length of these dispersed repeats was 23,456 bp, constituting 6.2% of the entire mitochondrial genome of *C. soulattri*. This observation underscores the prevalence of repeats in the mitochondrial genome.

### Analysis of plastid DNA insertion in the mitochondrial genome

Mitochondrial plastid DNAs (MTPTs) are specific DNA fragments originating from chloroplasts that are present within the mitochondrial genome^31^. We identified 23 fragments in the mitochondrial genome of *C. soulattri* that are highly homologous to the chloroplast genome. Figure 5 shows the locations of the identified MTPTs. Many complete chloroplast tRNA genes were found in MTPTs, including *trnV*-GAC, *trnS*-GGA, *trnD*-GUC, *trnW*-CCA, *trnN*-GUU, and *trnM*-CAU. Additionally, there is a chloroplast gene fragment, specifically *rrn16*, that is homologous to *rrn18* in the mitochondrial genome. Nevertheless, there were homologous fragments without gene annotations (Supplementary Table S6) that warrant further studies.

### Analysis of RNA editing site

We identified several RNA editing events in 30 PCGs from the mitochondrial genome of *C. soulattri*. Using Deepred-Mt with default parameters, we detected 345 potential RNA editing sites (Fig. 6). It is worth noting the substantial heterogeneity in the number of RNA editing sites among different genes. Among the PCGs, *nad4* exhibited the highest number, with 35 RNA editing sites, which surpass all other genes. This was followed by the *ccmB* gene, which displayed 33 RNA editing sites. Conversely, the *atp8*, *rpl10*, and *sdh4* genes displayed only one RNA editing site, whereas none of the *atp9* and *cob* genes. All RNA editing in the mitochondrial genome of *C. soulattri* was C-to-U editing (Supplementary Table S7).

### Analysis of genes under selective pressure

In this study, we calculated the Ka (non-synonymous substitution rate) and Ks (synonymous substitution rate) substitution ratio (Ka/Ks) of 29 shared PCGs of *C. soulattri* and *Passiflora edulis*. The majority of PCGs exhibited Ka/Ks ratios below 1, indicating the prevalence of purifying selection (Fig. 7 and Supplementary Table S8). This also suggests that a considerable portion of the mitochondrial genes underwent selective purification, which may contribute to the preservation of normal mitochondrial function. However, there were PCGs with Ka/Ks ratios greater than 1, particularly *atp4* and *ccmB*. These genes were associated with the mitochondrial respiratory chain. The elevated Ka/Ks ratios in these genes suggest that positive selection had occurred, leading to the emergence of beneficial traits during evolution.

### Phylogenetic analyses

To elucidate the phylogenetic position of *C. soulattri*, we obtained the mitochondrial genome sequences of 14 Malpighiales species and two non-Malpighiales species as outgroups from the NCBI GenBank (https://www.ncbi.nlm.nih.gov/genbank/). We used all core PCGs and one shared variable gene (*rps3*) for the phylogenetic analyses. Maximum likelihood (ML) and Bayesian inference (BI) phylogenetic analyses revealed the separation of 16 species into distinct taxonomic clusters based on their respective families (Fig. 8). Furthermore, the results indicated that *C. soulattri* is a sister taxon to *G. mangostana*, which is strongly supported by its high clade credibility (Shimodaira-Hasegawa-like approximate likelihood ratio test or SH-aLRT = 100, Ultrafast Bootstrap or UFBoot = 100, Posterior Probability or PP = 1.00). The phylogenetic tree also demonstrated concordance with the Angiosperm Phylogeny Group IV classification. This affirms the taxonomic position of *C. soulattri* in the family Calophyllaceae, which shares a close phylogenetic relationship with both Clusiaceae and Rhizophoraceae. Significantly, a substantial number of nodes exhibited high support values, indicating a high degree of confidence and reliability in phylogenetic analyses.

## Discussion

In this study, we sequenced and assembled the complete mitochondrial genome of *C. soulattri*, which was 378,262 bp long. Although we used only short reads to assemble the genome, we were still able to generate a computationally feasible mitochondrial genome assembly. It is also crucial to acknowledge the potential existence of isoforms and multi-chromosomal mitochondrial genome structure^13^. This structural diversity of the mitochondrial genome is evident in many medicinal and economically important plant species^32–42^.

The mitochondrial genome of *C. soulattri* comprises 24 core PCGs, similar to other typical angiosperms^43–45^, and six variable genes. It is also relatively larger than the mitochondrial genome of *G. mangostana* by 1.89%^20^. This disparity can be attributed to the presence of a greater number of ribosomal proteins and a succinate dehydrogenase gene in the mitochondrial genome of *C. soulattri* (Fig. 2). During evolution, it is possible that some PCGs underwent loss and were subsequently transferred to the nuclear genome^46,47^. This phenomenon has been observed in the mitochondrial genomes of some angiosperms, such as *Citrullus lanatus* and *Cucumis melo*^48^.

Repeat analysis of the *C. soulattri* mitochondrial genome revealed 194 SSRs. Among these, the majority were mononucleotide (A/T) repeats similar to those reported in *Gleditsia sinensis*, *Gentiana* spp., and *Camellia gigantocarpa*^49–51^. The prevalence of these repeats may be attributed to the low GC content of the mitochondrial genome. This finding is consistent with observations made in the mitochondrial genomes of *Phaseolus vulgaris* and *Pereskia aculeata*^32,39^. In addition, we reported a significant presence of dispersed repeats within the mitochondrial genome of *C. soulattri*. These dispersed repeats primarily consisted of forward and palindromic repeats. Interestingly, this finding is also similar to the mitochondrial genomes of *Saposhnikovia divaricata*, *Artemisia giraldii*, and *Momordica charantia*^52–54^.

Angiosperms exhibit significant inter-organelle communication of genetic material within their cells. This communication primarily involves the transfer of DNA from the chloroplasts to the mitochondrial genome^55^. Here, we discovered 23 MTPTs in the *C. soulattri* mitochondrial genome. Most of the MTPTs are tRNA genes. The existence of these chloroplast-derived tRNAs in the mitochondrial genome is likely due to horizontal gene transfer (HGT)^56^. Furthermore, MTPTs are also well reported in other angiosperms such as *Salvia miltiorrhiza*, *Taraxacum mangolicum*, and *Abelmoschus esculentus*^37,38,41^.

Eukaryotes usually employ a process called RNA editing to modify the coding regions of transcribed RNA by inserting, deleting, or converting nucleotide bases^57^. Extensive studies have demonstrated a wide prevalence of RNA editing in higher plant mitochondria^58^. RNA editing often plays a crucial role in gene expression in the mitochondrial genomes of plants^59^. Primarily, it involves a deamination reaction that converts cytosine (C) to uracil (U) at specific sites in most angiosperms^60^. RNA editing events in the mitochondrial genome have been reported in *Prunus salicina* and *S. officinalis*^36,37^. We documented 345 RNA editing events in the mitochondrial genome of *C. soulattri*. Significantly, our study revealed that RNA editing events generated the start codon of *nad1* and stop codon of *ccmFc* (Supplementary Table S3, Supplementary Table S7), which was also observed in *G. mangostana*^20^. The creation of new start and stop codons gives rise to proteins that exhibit high conservation and homology. This enhances gene expression in mitochondria^61^.

Analysis of Ka/Ks ratio yields valuable insights into the evolutionary dynamics of plant mitochondrial genes^62^. A Ka/Ks ratio of 1 signifies neutral mutation, whereas a Ka/Ks ratio $<1$ indicates negative or purifying selection. Conversely, a Ka/Ks ratio $>1$ suggests the influence of positive or diversifying selection. Here, most mitochondrial genes displayed a pronounced conservation pattern, indicative of neutral or negative selection. Nonetheless, our analysis revealed two specific genes, *atp4* and *ccmB*, exhibiting a Ka/Ks ratio greater than 1.0, suggesting the likelihood of positive selection during their evolutionary history. The *atp4* gene encodes a subunit of the F0 (F-type ATPase) sector and its protein product contributes to proton transmembrane transfer, which is fundamental to ATP synthesis. The F0 sector serves as a proton channel through which protons (H+) flow back into the mitochondrial matrix^63^. The F0 subunit encoded by *atp4* forms part of the proton channel, allowing protons to flow back into the matrix through the ATP synthase complex^64^. The *ccmB* gene encodes a *ccmB*-like mitochondrial protein involved in the biogenesis of c-type cytochromes, which are essential components of electron transfer processes. The *ccmB* gene product, a *ccmB*-like mitochondrial protein, plays a key role in the transportation of heme to the mitochondrion^65,66^. Ensuring the proper export and integration of heme into c-type cytochromes is vital for the optimal functioning of these electron transfer proteins. These two genes (*atp* and *ccmB*) may have undergone adaptive changes, potentially developing new functions. These adaptations may have occurred in response to environmental or physiological pressures, possibly contributing to stress resistance. In contrast, *atp9* experienced robust negative selection. The *atp9* gene encodes a subunit of the ATP synthase complex. The ATP9 protein plays a significant role in proton-transporting ATP synthase activity. Moreover, it is very important in the regulation of proton translocation during proton transfer within the ATP synthase complex^64^. The pronounced purification and selection of this gene are likely due to its integral biological function in plants. Without functional *atp9* genes and the ATP synthase complex, plants would be unable to efficiently generate ATP, leading to severe impairment of energy-dependent processes that are essential for their growth, development, and survival. These findings are consistent with observations in *Scuttelaria tsinyunensis*, where *atp4* and *ccmB* showed evidence of positive selection, whereas *atp9* exhibited strong purifying selection^41^. Furthermore, *atp4* and *ccmB* were also found to be under positive selection in *Salix wilsonii*^16^.

The mitochondrial genome of vascular plants exhibits a slow evolutionary rate, characterized by significantly low mutation rates^67^. This distinctive feature renders it a valuable tool for phylogenetic research, facilitating the study of the evolutionary relationships among plant species. Our current phylogenetic analysis reveals that *C. soulattri* is closely related to *G. mangostana* and *B. sexangula*, confirming previous findings using plastid and nuclear DNA sequences^15,68^.

## Conclusion

We successfully assembled and annotated the complete *C. soulattri* mitochondrial genome. The genome was circular and 378,262 bp in length, with a GC content of 43.97%. A detailed analysis of the genome provides valuable genetic resources for conducting phylogenetic studies on *C. soulattri*, and lays a solid foundation for future research on mitochondrial genomes within the Calophyllaceae family.

## Methods

### Plant material, total genomic DNA extraction, and sequencing

The fresh leaves of *C. soulattri* used in this study were collected from the forest trees germplasm of the Metallophytes Laboratory at the University of the Philippines, Los Baños, Laguna ($14^\circ 9'17''\,N,121^\circ 14'6.25''\,E$). A leaf specimen was submitted to the Jose Vera Santos Memorial Herbarium at the Institute of Biology, University of the Philippines, Diliman, Quezon City and compared with accession number 14288. Leaf samples were quickly frozen in liquid nitrogen and then stored at –$80^{\circ }\hbox {C}$ refrigerator prior to DNA extraction. High-quality total genomic DNA was extracted using a previously described protocol^15^. The processed total gDNA samples were submitted to a local distributor for outsourced next-generation sequencing to Macrogen, Inc. (Seoul, South Korea). Briefly, paired-end libraries were generated using TruSeq$\circledR$ DNA Nano Library Prep kit and sequenced using the Illumina HiSeq 2500 platform (San Diego, California, USA). The sequencing data were deposited in GenBank (https://www.ncbi.nlm.nih.gov/) under BioProject ID PRJNA891016.

### Mitochondrial genome assembly and annotation

The mitochondrial genome of *C. soulattri* was assembled using GetOrganelle v1.7.6.1^69^ using the following parameters: -R 20 -k 21,45,65,86,105 -p 1000000 -F embplant_mt. In addition, NOVOPlasty v4.3.1^70^ was used to generate artificial long-reads or scaffolds. Briefly, makeblastdb was used to construct a database of the assembly generated by NOVOPlasty and then using the BLASTn program, contigs produced by GetOrganelle were searched against the database. Assessment and manual editing of the assembly to obtain a complete circular contig were done with the aid of Bandage v0.8.1^71^. The assembled *C. soulattri* mitogenome was annotated using IPGMA (http://www.1kmpg.cn/mga). The assembled mitogenome of *C. soulattri* was also BLAST-searched against protein-coding genes (PCGs) and ribosomal RNA (rRNA) genes deposited at NCBI to verify the annotation results. Subsequently, manual editing and correction of erroneous annotations were performed using Apollo software v1.11.8^72^. The physical circular mitochondrial genome map of *C. soulattri* was generated using Organellar Genome DRAW (OGDRAW) v1.3.1 program with default parameters^73^. The mitochondrial genome sequence was deposited in GenBank through BankIt (https://www.ncbi.nlm.nih.gov/WebSub/) and obtained the accession number OQ721861 or NC_079842.1.

### Analysis of codon usage and selection pressure of mitochondrial protein-coding genes

We extracted the protein-coding genes (PCGs) using PhyloSuite v1.2.3^74^ to parse the GenBank format file of *C. soulattri* mitochondrial genome. CodonW v1.4.2 (https://codonw.sourceforge.net/) was used to analyze the codon usage of mitochondrial PCGs by calculating RSCU values. The Ka/Ks_Calculator v2.0^75^ with the MLWL method was used to calculate the Ka/Ks of shared mitochondrial PCGs of *C. soulattri* and *P. edulis* (Accession No. NC_050950) in the Passifloraceae family of the order Malpighiales was used as an outgroup.

### Analysis of repeat elements

Simple sequence repeats (SSRs) were detected in the mitochondrial genome of *C. soulattri* using MISA-web (https://www.web-blast.ipk-gatersleben.de/misa/)^76^ with the following parameters: minimum number of repeats of mono-, di-, tri-, tetra-, penta-, and hexanucleotides were set as 8, 5, 4, 3, 3, and 3, respectively. For long repeat sequences, we used RePuter (https://www.bibiserv.cebitec.uni-bielefeld.de/reputer)^77^ to calculate forward, reverse, palindromic, and complementary repeat sequences with the following parameters: hamming distance of 3 and minimal repeat size of 30 bp, and e-value was limited to less than $1 \times 10^{-5}$.

### Analysis of mitochondrial plastic DNAs (MTPTs)

To identify DNA fragments that may have been transferred from the chloroplast genome, we downloaded the plastome of *C. soulattri* (Accession No. NC_068749) and compare it with the mitochondrial genome using BLAST in TBtools^78^ using the following parameters: e-value = $1 \times 10^{-5}$, number of hits = 50000, and number of aligns = 25000. Then, we visualized the distribution of MTPTs using Circos plot implemented in TBtools^79^.

### Analysis of RNA editing sites in the mitochondrial genome

We used Deepred-Mt^80^ in predicting C-to-U RNA editing based on the convolutional neural network (CNN) model. All protein-coding genes of *C. soulattri* were extracted from the mitochondrial genome and used for prediction. Results with probability values greater than 0.9 were selected for analysis.

### Phylogenetic analyses

We retrieved the mitochondrial genomes of 14 closely related species and two non-Malpighiales species on NCBI (https://www.ncbi.nlm.nih.gov/) nucleotide database. A total of 26 protein-coding genes that are present in all these mitochondrial genomes were identified and extracted using PhyloSuite v1.2.3^74^. Then, the sequences of each gene were aligned using MAFFT v7.471^81^ and concatenated into a data matrix. Subsequently, ModelFinder^82^ was used to find the best-fit substitution model and best partitioning scheme. Phylogenetic analyses were conducted using maximum likelihood (ML) and Bayesian inference (BI). The ML tree was constructed using IQ-TREE v2.2.0^83^. Furthermore, the Shimodaira-Hasegawa-like approximate likelihood ratio test (SH-aLRT)^84^ with 1000 replicates and ultrafast bootstrap (UFBoot)^85^ with 5000 replicates were used to assess clade support. The BI phylogenetic analysis was performed using MrBayes v3.2.7a^86^ with two independent runs using four MCMC chains. The trees were sampled every 1000 generations for 10,000,000 generations. Finally, all results were uploaded to iTOL viewer (https://www.itol.embl.de/)^87^ for editing and tree visualization.

### Ethics approval and consent to participate

This study has obtained permission to collect *C. soulattri* through the Department of Environment and Natural Resources (DENR BMB Gratuitous Permit No. 299, 2019). We also conducted the study in accordance with relevant institutional, national and international guidelines and legislation.

### Supplementary Information

Supplementary Information 1.

Supplementary Information 2.

## Data Availability

The complete mitochondrial genome sequence data that support the findings of this study are openly available in GenBank of NCBI database at (https://www.ncbi.nlm.nih.gov/) under the accession number OQ721861/NC_079842. The associated BioProject, SRA, and BioSample numbers are PRJNA891016, SRR22031315-SRR24822418, and SAMN31427486, respectively.
