# Supplementary material for: The first mitochondrial genome of Calophyllum soulattri Burm.f
Source: Sci Rep. 2024 Mar 1;14:5112. doi: 10.1038/s41598-024-55016-6 (PMC10907642; doi:10.1038/s41598-024-55016-6)
Supplement: Supplementary file 1 — Supplementary Information 1. [file 41598_2024_55016_MOESM1_ESM.pdf]

The First Mitochondrial Genome of *Calophyllum soulattri* Burm. f.

Charles Anthon E. Cadorna[1], Dexter G. Pahayo[1], and Jessica D. Rey[1]

[1]Plant Molecular Phylogenetics Laboratory, Institute of Biology, College of Science, University of the Philippines, Diliman, Quezon City 1101, Philippines

Supplementary Figures

Figure S1. The assembly steps of *C. soulattri* mitochondrial genome

Figure S2. The genes in *C. soulattri* mitochondrial genome with introns and their corresponding type of splicing.

Figure S3. A comprehensive circular mitochondrial genome map of *C. soulattri*.

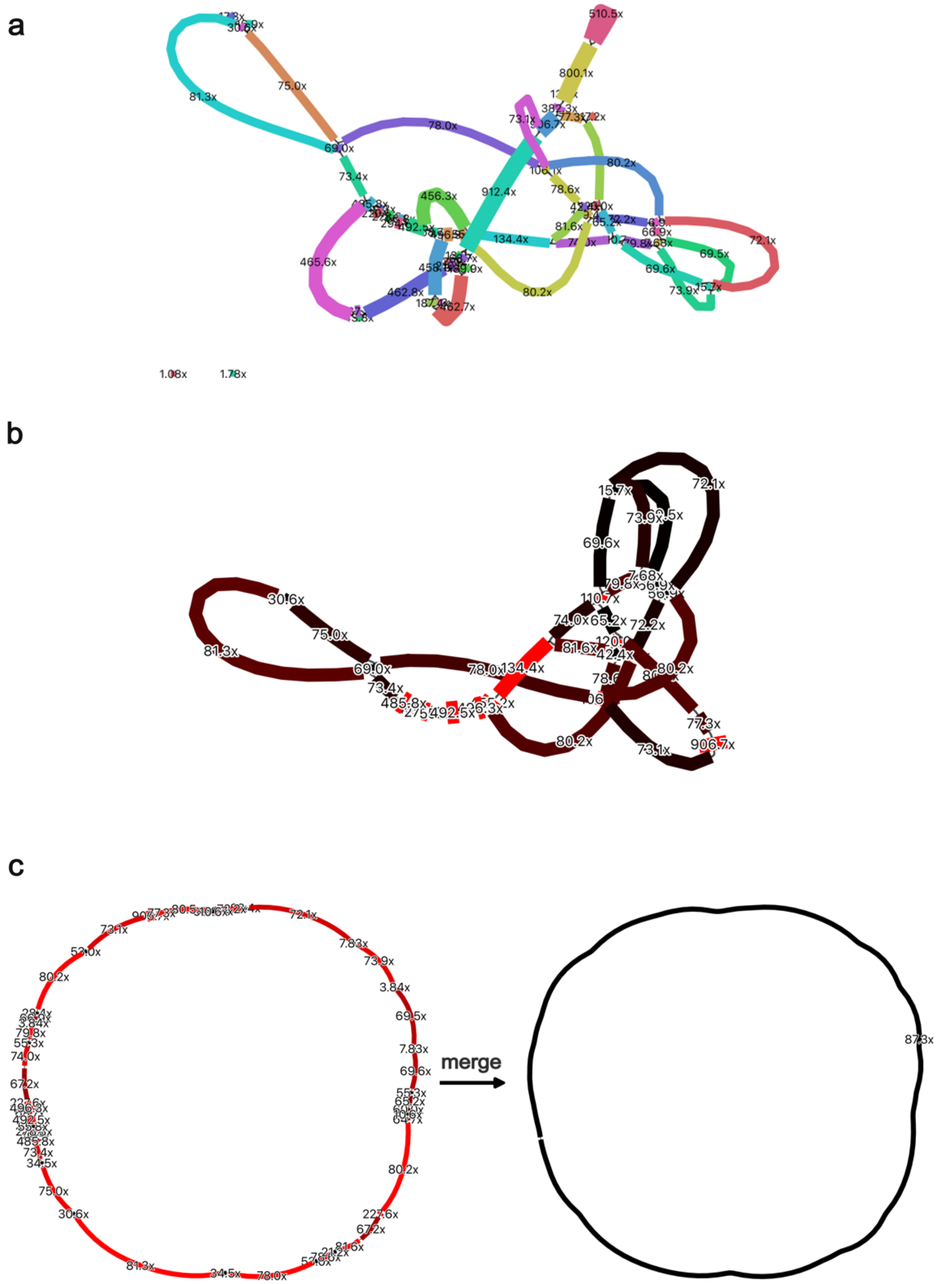

Figure S1. The assembly steps of *C. soulattri* mitochondrial genome. (**a**) The initial assembly graph of *C. soulattri* mitochondrial genome which was obtained from Illumina short reads using GetOrganelle. The assembly resulted in a complex network structure with some chloroplast sequences inserted as seen by the depth of coverage (**b**) The assembly graph of *C. soulattri* after removal of plastid sequences based on level of coverage. (**c**) The circular graph of *C. soulattri* mitochondrial genome after confirming the true path of duplicated regions using scaffolds generated using NOVOPlasty. All possible nodes where then merged.

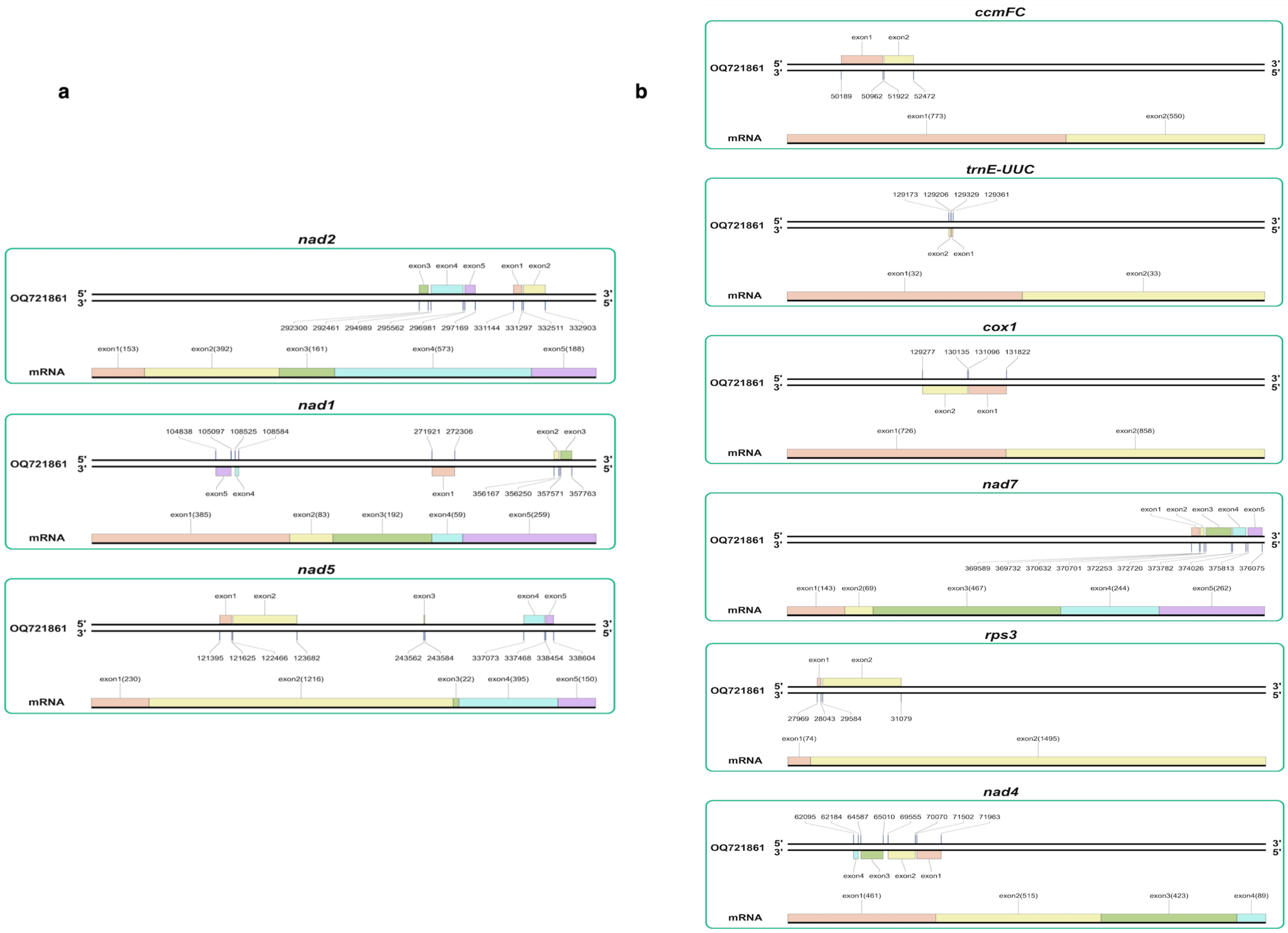

Figure S2. The genes in *C. soulattri* mitochondrial genome with introns and their corresponding type of splicing. (**a**) *trans*- and **(b**) *cis*-splicing genes in the mitochondrial genome of *C. soulattri*.

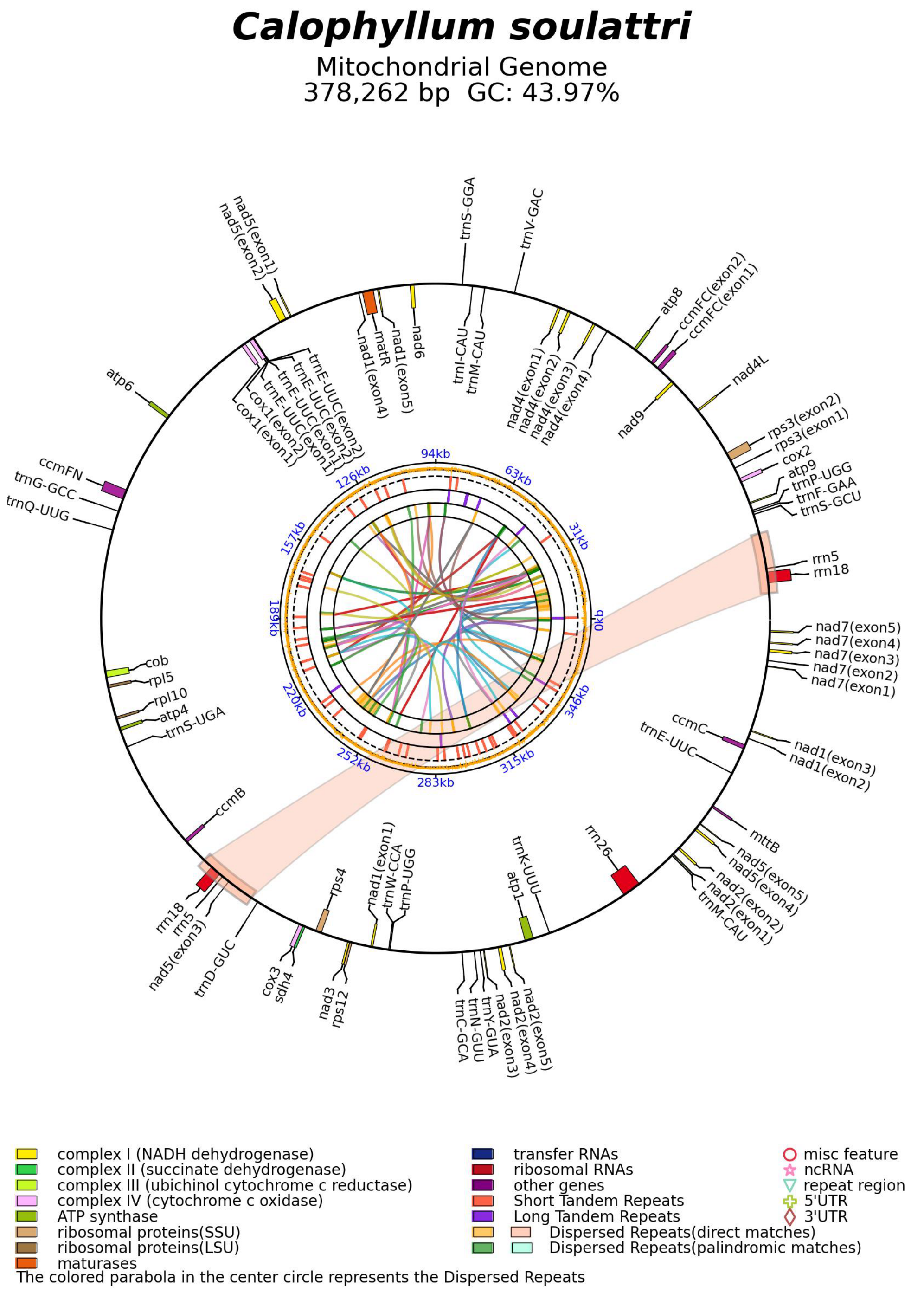

Figure S3. A comprehensive circular mitochondrial genome map of *C. soulattri*. The links in the innermost circles represent dispersed repeats. Green and yellow lines represent the palindrome and forward repeats, respectively. Bars on the second and third circles represent tandem repeats, and SSRs, respectively. The line graph inside the fourth circle represents the GC content. Genes inside the outermost circle were on the negative strand, whereas those on the outside were on the positive strand. The colors correspond to the different functional categories shown in the legend. The circular map was drawn using OGView (http://www.1kmpg.cn/pmgview).
