# Supplementary material for: The first mitochondrial genome of Calophyllum soulattri Burm.f
Source: Sci Rep. 2024 Mar 1;14:5112. doi: 10.1038/s41598-024-55016-6 (PMC10907642; doi:10.1038/s41598-024-55016-6)
Supplement: Supplementary file 2 — Supplementary Information 2. [file 41598_2024_55016_MOESM2_ESM.pdf]

The First Mitochondrial Genome of *Calophyllum soulattri* Burm. f.

Charles Anthon E. Cadorna[1], Dexter G. Pahayo[1], and Jessica D. Rey[1]

[1]Plant Molecular Phylogenetics Laboratory, Institute of Biology, College of Science, University of the Philippines, Diliman, Quezon City 1101, Philippines

Supplementary Tables

Table S1. Sequencing data for *C. soulattri* mitochondrial genome.

Table S2. Length of protein-coding genes in the mitochondrial genome of some species in the order Malpighiales.

Table S3. Start codon and stop codon in the protein-coding genes of *C. soulattri* mitochondrial genome.

Table S4. Codon usage of the PCGs of *C. soulattri* mitochondrial genome.

Table S5. Dispersed repeats (≥30 bp) in the mitochondrial genome of *C. soulattri*.

Table S6. The homologous DNA fragment identified among the mitochondrial and chloroplast genomes of *C. soulattri*.

Table S7. RNA editing events identified in the mitochondrial PCGs of *C. soulattri*.

Table S8. Ka/Ks ratios of PCGs in the mitochondrial genome of *C. soulattri.*

Table S1. Sequencing data for *C. soulattri* mitochondrial genome.

Sample ID	Total bases (bp)	Total reads	GC (%)	AT (%)	Q20 (%)	Q30 (%)
CsHMWgDNA1	2,758,456,248	27,311,448	35.9	64.1	96.4	94.0
CsHMWgDNA2	2,320,982,020	22,980,020	36.0	64.0	96.6	94.2

Table S2. Length of protein-coding genes in the mitochondrial genome of some species in the order Malpighiales.

Gene	*C. soulattri*	*S. wilsonii*	*S. triandra*	*S. dunnii*	*S. brachista*	*B. sexangula*	*P. edulis*	*P. davidiana*	*S. purpurea*	*S. suchowensis*	*P. tremula*	*S. arbutifolia*	*G. mangostana*	*P. rotundifolia*
atp1	1527	1524	1524	1524	1524	1536	1530	1524	1524	1596	1524	1524	1512	1524
atp4	576	597	597	597	597	612	597	597	597	597	597	597	612	597
atp6	720	714	714	714	714	801	1158	714	714	714	714	714	768	714
atp8	480	474	474	474	474	480	489	474	474	474	474	474	474	474
atp9	225	225	225	225	225	225	252	225	225	225	225	225	225	225
ccmB	621	615	615	615	615	615	621	615	615	615	615	615	621	615
ccmC	753	753	753	753	753	828	753	753	753	753	753	753	753	753
ccmFc	1323	1362	1362	1368	1362	1317	1311	1362	1356	1362	1362	1362	1341	1362
ccmFn	1734	1725	1725	1725	1725	1731	1749	1731	1725	1725	1731	1725	1722	1731
cob	1182	1182	1182	1182	1182	1182	1173	1182	1182	1182	1182	1182	1182	1182
cox1	1584	1584	1584	1584	1584	1584	1833	1584	1584	1584	1584	1584	1575	1584
cox2	700	675	675	675	675	783	777	675	675	675	675	675	795	675
cox3	798	798	798	798	798	798	798	798	798	798	798	798	798	798
matR	1965	1944	1944	1944	1944	1950	1971	1944	1944	1944	1944	1944	1959	1944
mttB	372	786	786	729	729	846	852	786	786	786	786	786	780	786
nad1	978	894	894	888	888	828	888	888	888	888	888	894	978	888
nad2	1467	1461	1461	1461	1461	1467	1467	1461	1461	1461	1461	1461	1467	1461
nad3	357	357	357	357	357	357	357	357	357	357	357	357	357	357
nad4	1488	1488	1488	1488	1488	1488	1488	1488	1488	1488	1488	1488	1488	1488
nad4L	303	303	303	303	303	303	303	303	303	303	303	303	303	303
nad5	2013	2004	2004	1983	1983	1992	1992	2001	2004	2004	2001	1983	2013	2001
nad6	693	630	630	630	630	663	615	630	630	630	630	630	618	630
nad7	1185	1185	1185	1185	1185	1185	1185	1185	1185	1185	1185	1185	1185	1185
nad9	573	573	573	573	573	573	573	573	573	573	573	573	573	573
rpl10	447	489	489	489	-	-	465	489	459	489	489	489	441	489
rpl16	-	411	411	162	162	-	249	-	411	411	-	411	447	-
rpl2	-	1029	1023	1032	1032	-	-	1008	1032	1032	1008	1029	-	1008
rpl23	-	-	-	-	-	-	-	-	-	-	-	-	282	-
rpl5	498	-	-	-	-	561	561	-	-	-	-	-	615	-
rps1	-	690	690	690	663	723	669	636	-	-	636	690	-	636
rps10	-	-	-	-	-	-	-	-	-	-	-	-	-	-
rps12	378	378	378	378	378	378	378	378	378	390	378	378	-	378
rps14	-	-	-	141	141	-	-	246	-	-	246	-	-	246
rps19	-	-	-	-	-	-	285	-	-	-	-	-	-	-
rps3	1569	1644	1644	1656	1656	1752	1689	1644	1644	1644	1644	1644	1485	1644
rps4	1053	969	969	969	969	-	954	969	969	969	969	969	1026	969
rps7	-	-	447	273	336	-	-	447	447	447	447	447	-	447
sdh3	-	-	-	-	-	222	288	-	-	-	-	-	-	-
sdh4	402	396	396	396	396	399	-	396	396	396	396	396	-	396

Table S3. Start codon and stop codon in the protein-coding genes of *C. soulattri* mitochondrial genome.

Genes	Length (bp)	Start Codon	Stop Codon
atp1	1527	ATG	TGA
atp4	576	ATG	TAG
atp6	720	ATG	TAA
atp8	480	ATG	TAA
atp9	225	ATG	TAA
ccmB	621	ATG	TGA
ccmC	753	ATG	TGA
ccmFc	1323	ATG	CGA
ccmFn	1734	ATG	TGA
cob	1182	ATG	TAA
cox1	1584	ATG	TAA
cox2	700	ATG	Not determined
cox3	798	ATG	TGA
matR	1965	ATG	TAG
mttB	372	ATG	TAA
nad1	978	ACG	TAA
nad2	1467	ATG	TAA
nad3	357	ATG	TAA
nad4	1488	ATG	TGA
nad4L	303	ATG	TAA
nad5	2013	ATG	TAA
nad6	693	ATG	TAA
nad7	1185	ATG	TAG
nad9	573	ATG	TAA
rpl10	447	ATG	TAA
rpl5	498	ATG	Not determined
rps12	378	ATG	TGA
rps3	1569	ATG	TGA
rps4	1053	ATG	TAA
sdh4	402	ATG	TAG

Table S4. Codon usage of the PCGs of *C. soulattri* mitochondrial genome.

Codon	Count	RSCU	Codon	Count	RSCU	Codon	Count	RSCU	Codon	Count	RSCU
UUU(F)	393	1.19	UCU(S)	195	1.41	UAU(Y)	233	1.53	UGU(C)	86	1.27
UUC(F)	268	0.81	UCC(S)	129	0.93	UAC(Y)	72	0.47	UGC(C)	49	0.73
UUA(L)	270	1.51	UCA(S)	161	1.17	UAA(*)	15	1.67	UGA(*)	8	0.89
UUG(L)	203	1.14	UCG(S)	108	0.78	UAG(*)	4	0.44	UGG(W)	147	1
CUU(L)	224	1.25	CCU(P)	181	1.56	CAU(H)	177	1.56	CGU(R)	110	1.19
CUC(L)	117	0.65	CCC(P)	90	0.78	CAC(H)	50	0.44	CGC(R)	58	0.63
CUA(L)	169	0.95	CCA(P)	126	1.09	CAA(Q)	192	1.54	CGA(R)	123	1.33
CUG(L)	89	0.5	CCG(P)	67	0.58	CAG(Q)	58	0.46	CGG(R)	63	0.68
AUU(I)	315	1.29	ACU(T)	166	1.43	AAU(N)	212	1.36	AGU(S)	150	1.09
AUC(I)	213	0.87	ACC(T)	125	1.08	AAC(N)	100	0.64	AGC(S)	85	0.62
AUA(I)	207	0.84	ACA(T)	118	1.02	AAA(K)	248	1.22	AGA(R)	138	1.49
AUG(M)	262	1	ACG(T)	55	0.47	AAG(K)	160	0.78	AGG(R)	64	0.69
GUU(V)	183	1.25	GCU(A)	227	1.57	GAU(D)	204	1.37	GGU(G)	207	1.3
GUC(V)	111	0.76	GCC(A)	133	0.92	GAC(D)	93	0.63	GGC(G)	79	0.5
GUA(V)	175	1.2	GCA(A)	149	1.03	GAA(E)	257	1.38	GGA(G)	239	1.5
GUG(V)	115	0.79	GCG(A)	69	0.48	GAG(E)	116	0.62	GGG(G)	111	0.7

*NOTE: RSCU refers to relative synonymous codon usage. Asterisk(*) refers to the termination codon.

Table S4. Statistics on SSRs in the mitochondrial genome of *C. soulattri.*

ID	SSR nr.	SSR type	SSR	Size	Start	End
Calophyllum soulattri	1	p1	(A)8	8	457	464
Calophyllum soulattri	2	p1	(G)8	8	6959	6966
Calophyllum soulattri	3	p1	(A)9	9	10997	11005
Calophyllum soulattri	4	p1	(T)9	9	11590	11598
Calophyllum soulattri	5	p4	(AGAT)3	12	15406	15417
Calophyllum soulattri	6	p1	(T)8	8	20257	20264
Calophyllum soulattri	7	p1	(T)8	8	20405	20412
Calophyllum soulattri	8	p1	(A)8	8	20974	20981
Calophyllum soulattri	9	p1	(A)8	8	21968	21975
Calophyllum soulattri	10	p1	(A)8	8	25446	25453
Calophyllum soulattri	11	p1	(A)8	8	25971	25978
Calophyllum soulattri	12	p3	(TCC)4	12	26630	26641
Calophyllum soulattri	13	p3	(TTG)4	12	27731	27742
Calophyllum soulattri	14	p1	(A)9	9	28100	28108
Calophyllum soulattri	15	p1	(A)8	8	29948	29955
Calophyllum soulattri	16	p1	(T)8	8	30429	30436
Calophyllum soulattri	17	p2	(GA)5	10	34694	34703
Calophyllum soulattri	18	p1	(T)9	9	35087	35095
Calophyllum soulattri	19	p1	(A)9	9	36544	36552
Calophyllum soulattri	20	p1	(A)10	10	37553	37562
Calophyllum soulattri	21	p1	(T)9	9	38496	38504
Calophyllum soulattri	22	p1	(T)8	8	47343	47350
Calophyllum soulattri	23	p4	(GGAA)3	12	49075	49086
Calophyllum soulattri	24	p1	(T)8	8	52377	52384
Calophyllum soulattri	25	p1	(A)8	8	52709	52716
Calophyllum soulattri	26	p1	(T)9	9	54860	54868
Calophyllum soulattri	27	p1	(T)9	9	55317	55325
Calophyllum soulattri	28	p1	(T)9	9	58983	58991
Calophyllum soulattri	29	p1	(T)8	8	59728	59735
Calophyllum soulattri	30	p1	(G)8	8	60996	61003
Calophyllum soulattri	31	p1	(T)8	8	62772	62779
Calophyllum soulattri	32	p1	(A)8	8	63777	63784
Calophyllum soulattri	33	p1	(T)8	8	65111	65118
Calophyllum soulattri	34	p1	(A)8	8	71456	71463
Calophyllum soulattri	35	p1	(A)8	8	76520	76527
Calophyllum soulattri	36	p1	(T)8	8	80933	80940
Calophyllum soulattri	37	p1	(A)8	8	82616	82623
Calophyllum soulattri	38	p3	(CTT)5	15	85127	85141
Calophyllum soulattri	39	p2	(CT)5	10	85408	85417
Calophyllum soulattri	40	p1	(A)8	8	85727	85734
Calophyllum soulattri	41	p4	(GAAT)3	12	86217	86228
Calophyllum soulattri	42	p1	(A)10	10	88056	88065
Calophyllum soulattri	43	p1	(A)9	9	89555	89563
Calophyllum soulattri	44	p4	(CTGG)3	12	93395	93406
Calophyllum soulattri	45	p1	(T)9	9	94649	94657
Calophyllum soulattri	46	p5	(TGAAG)3	15	95554	95568
Calophyllum soulattri	47	p3	(TAA)4	12	96844	96855
Calophyllum soulattri	48	p1	(A)9	9	97264	97272
Calophyllum soulattri	49	p1	(T)8	8	100200	100207
Calophyllum soulattri	50	p3	(AAT)4	12	100763	100774
Calophyllum soulattri	51	p1	(T)9	9	102554	102562
Calophyllum soulattri	52	p1	(A)8	8	105681	105688
Calophyllum soulattri	53	p1	(A)10	10	108555	108564
Calophyllum soulattri	54	p1	(A)9	9	109738	109746
Calophyllum soulattri	55	p2	(CA)5	10	110716	110725
Calophyllum soulattri	56	p1	(A)8	8	110993	111000
Calophyllum soulattri	57	p1	(T)8	8	115984	115991
Calophyllum soulattri	58	p1	(A)11	11	116774	116784
Calophyllum soulattri	59	p1	(T)8	8	119787	119794
Calophyllum soulattri	60	p1	(A)8	8	119988	119995
Calophyllum soulattri	61	p5	(TACTA)3	15	121209	121223
Calophyllum soulattri	62	p1	(G)8	8	121755	121762
Calophyllum soulattri	63	p2	(CT)5	10	124681	124690
Calophyllum soulattri	64	p1	(A)16	16	128254	128269
Calophyllum soulattri	65	p2	(AG)5	10	131853	131862
Calophyllum soulattri	66	p3	(ACT)4	12	134587	134598
Calophyllum soulattri	67	p2	(AT)5	10	138975	138984
Calophyllum soulattri	68	p1	(A)8	8	143502	143509
Calophyllum soulattri	69	p3	(AGA)4	12	144081	144092

Calophyllum soulattri	70	p3	(AAG)4	12	145976	145987
Calophyllum soulattri	71	p1	(A)8	8	147084	147091
Calophyllum soulattri	72	p1	(A)9	9	150698	150706
Calophyllum soulattri	73	p1	(T)8	8	150811	150818
Calophyllum soulattri	74	p1	(T)10	10	151752	151761
Calophyllum soulattri	75	p4	(GGAG)3	12	156182	156193
Calophyllum soulattri	76	p1	(A)9	9	156942	156950
Calophyllum soulattri	77	p1	(T)9	9	167915	167923
Calophyllum soulattri	78	p1	(A)10	10	170893	170902
Calophyllum soulattri	79	p1	(T)11	11	172107	172117
Calophyllum soulattri	80	p4	(TCAT)3	12	172474	172485
Calophyllum soulattri	81	p2	(AT)5	10	175311	175320
Calophyllum soulattri	82	p1	(A)8	8	176061	176068
Calophyllum soulattri	83	p1	(T)8	8	178893	178900
Calophyllum soulattri	84	p4	(AAGC)3	12	180123	180134
Calophyllum soulattri	85	p2	(TC)5	10	180495	180504
Calophyllum soulattri	86	p1	(A)9	9	183212	183220
Calophyllum soulattri	87	p2	(AG)5	10	185466	185475
Calophyllum soulattri	88	p1	(A)10	10	188442	188451
Calophyllum soulattri	89	p1	(T)8	8	192345	192352
Calophyllum soulattri	90	p3	(CTT)4	12	192485	192496
Calophyllum soulattri	91	p3	(GAA)4	12	194137	194148
Calophyllum soulattri	92	p1	(T)9	9	195468	195476
Calophyllum soulattri	93	p1	(T)10	10	198318	198327
Calophyllum soulattri	94	p4	(GGAA)3	12	200739	200750
Calophyllum soulattri	95	p1	(T)13	13	201075	201087
Calophyllum soulattri	96	p1	(A)9	9	201785	201793
Calophyllum soulattri	97	p2	(TA)5	10	202153	202162
Calophyllum soulattri	98	p1	(T)10	10	208162	208171
Calophyllum soulattri	99	p1	(T)10	10	212048	212057
Calophyllum soulattri	100	p1	(A)8	8	214197	214204
Calophyllum soulattri	101	p1	(A)9	9	216782	216790
Calophyllum soulattri	102	p3	(CTT)4	12	221532	221543
Calophyllum soulattri	103	p2	(GA)5	10	223026	223035
Calophyllum soulattri	104	p1	(A)10	10	227500	227509
Calophyllum soulattri	105	p1	(A)14	14	229804	229817
Calophyllum soulattri	106	p3	(CTT)4	12	230940	230951
Calophyllum soulattri	107	p1	(T)11	11	235276	235286
Calophyllum soulattri	108	p1	(A)8	8	236960	236967
Calophyllum soulattri	109	p1	(G)8	8	240077	240084
Calophyllum soulattri	110	p4	(GGCG)3	12	241966	241977
Calophyllum soulattri	111	p1	(A)9	9	244115	244123
Calophyllum soulattri	112	p1	(T)9	9	244708	244716
Calophyllum soulattri	113	p4	(AGAT)3	12	248524	248535
Calophyllum soulattri	114	p1	(T)10	10	249280	249289
Calophyllum soulattri	115	p1	(A)13	13	249497	249509
Calophyllum soulattri	116	p1	(T)9	9	249738	249746
Calophyllum soulattri	117	p1	(A)10	10	249858	249867
Calophyllum soulattri	118	p4	(AAGA)3	12	250640	250651
Calophyllum soulattri	119	p1	(T)8	8	258738	258745
Calophyllum soulattri	120	p3	(CTT)4	12	260780	260791
Calophyllum soulattri	121	p1	(A)16	16	261460	261475
Calophyllum soulattri	122	p1	(T)18	18	262020	262037
Calophyllum soulattri	123	p1	(A)8	8	263028	263035
Calophyllum soulattri	124	p1	(A)8	8	264853	264860
Calophyllum soulattri	125	p1	(A)9	9	265107	265115
Calophyllum soulattri	126	p5	(CGGAA)3	15	265271	265285
Calophyllum soulattri	127	p1	(T)11	11	267641	267651
Calophyllum soulattri	128	p1	(T)13	13	271015	271027
Calophyllum soulattri	129	p1	(A)8	8	274048	274055
Calophyllum soulattri	130	p3	(AAG)4	12	277125	277136
Calophyllum soulattri	131	p1	(A)8	8	280067	280074
Calophyllum soulattri	132	p1	(A)9	9	281210	281218
Calophyllum soulattri	133	p1	(A)8	8	282056	282063
Calophyllum soulattri	134	p1	(A)10	10	284610	284619
Calophyllum soulattri	135	p4	(ATAG)3	12	286757	286768
Calophyllum soulattri	136	p1	(A)18	18	287619	287636
Calophyllum soulattri	137	p4	(TTCA)3	12	291135	291146
Calophyllum soulattri	138	p1	(T)8	8	292116	292123
Calophyllum soulattri	139	p1	(A)10	10	293614	293623
Calophyllum soulattri	140	p1	(T)8	8	293852	293859

Calophyllum soulattri	141	p1	(T)8	8	298127	298134
Calophyllum soulattri	142	p1	(T)10	10	298376	298385
Calophyllum soulattri	143	p1	(T)8	8	300018	300025
Calophyllum soulattri	144	p1	(A)9	9	302412	302420
Calophyllum soulattri	145	p1	(T)11	11	302704	302714
Calophyllum soulattri	146	p1	(A)8	8	303789	303796
Calophyllum soulattri	147	p1	(T)11	11	305722	305732
Calophyllum soulattri	148	p2	(CT)5	10	306773	306782
Calophyllum soulattri	149	p3	(TAG)4	12	307076	307087
Calophyllum soulattri	150	p1	(T)11	11	309196	309206
Calophyllum soulattri	151	p2	(AG)5	10	310234	310243
Calophyllum soulattri	152	p2	(GA)6	12	310480	310491
Calophyllum soulattri	153	p4	(GGAA)3	12	314269	314280
Calophyllum soulattri	154	p1	(T)8	8	315287	315294
Calophyllum soulattri	155	p1	(T)9	9	316094	316102
Calophyllum soulattri	156	p1	(A)9	9	316430	316438
Calophyllum soulattri	157	p4	(TTCG)3	12	318454	318465
Calophyllum soulattri	158	p2	(TC)5	10	318927	318936
Calophyllum soulattri	159	p1	(A)15	15	319040	319054
Calophyllum soulattri	160	p1	(A)14	14	319539	319552
Calophyllum soulattri	161	p1	(C)10	10	320377	320386
Calophyllum soulattri	162	p1	(A)8	8	321326	321333
Calophyllum soulattri	163	p1	(T)10	10	323274	323283
Calophyllum soulattri	164	p4	(AGTA)3	12	324268	324279
Calophyllum soulattri	165	p1	(A)9	9	324389	324397
Calophyllum soulattri	166	p1	(A)9	9	334400	334408
Calophyllum soulattri	167	p1	(A)10	10	334656	334665
Calophyllum soulattri	168	p1	(T)9	9	336176	336184
Calophyllum soulattri	169	p1	(A)8	8	336540	336547
Calophyllum soulattri	170	p2	(TA)5	10	337778	337787
Calophyllum soulattri	171	p1	(T)10	10	338722	338731
Calophyllum soulattri	172	p2	(AT)5	10	343390	343399
Calophyllum soulattri	173	p4	(TTCT)3	12	343554	343565
Calophyllum soulattri	174	p1	(A)8	8	344261	344268
Calophyllum soulattri	175	p5	(TAATA)5	25	345687	345711
Calophyllum soulattri	176	p1	(T)8	8	347407	347414
Calophyllum soulattri	177	p1	(A)10	10	348229	348238
Calophyllum soulattri	178	p1	(A)9	9	351326	351334
Calophyllum soulattri	179	p1	(T)9	9	352229	352237
Calophyllum soulattri	180	p1	(A)9	9	357039	357047
Calophyllum soulattri	181	p1	(A)9	9	358050	358058
Calophyllum soulattri	182	p1	(A)9	9	358188	358196
Calophyllum soulattri	183	p1	(G)9	9	358382	358390
Calophyllum soulattri	184	p4	(CTAG)3	12	359094	359105
Calophyllum soulattri	185	p1	(T)8	8	359324	359331
Calophyllum soulattri	186	p1	(T)9	9	365699	365707
Calophyllum soulattri	187	p4	(AAGA)3	12	367341	367352
Calophyllum soulattri	188	p4	(AGAA)3	12	367731	367742
Calophyllum soulattri	189	p1	(C)8	8	368142	368149
Calophyllum soulattri	190	p5	(TTAGT)3	15	368356	368370
Calophyllum soulattri	191	p1	(T)8	8	368582	368589
Calophyllum soulattri	192	p1	(T)10	10	371359	371368
Calophyllum soulattri	193	p1	(T)9	9	376518	376526
Calophyllum soulattri	194	p1	(T)9	9	377098	377106

Table S5. Dispersed repeats (≥30 bp) identified in the mitochondrial genome of *C. soulattri*.

Species	ID	Repeat Types	Repeat Length (bp)	Repeat Units I (Start)	Repeat Units I (End)	Repeat Units II (Start)	Repeat Units II (End)	Repeat Interval	E-value
Calophyllum soulattri	R1	F	10913	4561	15473	237679	248591	0	0.00E+00
Calophyllum soulattri	R2	F	1140	97419	98558	203899	205038	0	0.00E+00
Calophyllum soulattri	R3	P	457	22673	23129	199766	200222	0	2.91E-265
Calophyllum soulattri	R4	P	247	55026	55272	262489	262735	0	7.87E-139
Calophyllum soulattri	R5	F	226	293235	293460	371791	372016	-1	2.35E-123
Calophyllum soulattri	R6	F	128	28936	29063	105483	105610	0	3.48E-67
Calophyllum soulattri	R7	F	108	97937	98044	257298	257405	0	3.82E-55
Calophyllum soulattri	R8	F	108	204417	204524	257298	257405	0	3.82E-55
Calophyllum soulattri	R9	P	96	144477	144572	186107	186202	0	6.41E-48
Calophyllum soulattri	R10	P	104	45089	45192	343171	343274	-3	4.81E-46
Calophyllum soulattri	R11	F	90	27854	27943	164350	164439	0	2.63E-44
Calophyllum soulattri	R12	F	100	20055	20154	121343	121442	-3	1.09E-43
Calophyllum soulattri	R13	F	85	0	84	378177	378261	0	2.69E-41
Calophyllum soulattri	R14	F	73	27401	27473	186806	186878	0	4.51E-34
Calophyllum soulattri	R15	P	81	89246	89326	377137	377217	-3	1.59E-32
Calophyllum soulattri	R16	P	79	55773	55851	209746	209824	-3	2.35E-31
Calophyllum soulattri	R17	F	78	20084	20161	121372	121449	-3	9.05E-31
Calophyllum soulattri	R18	F	65	34308	34372	201816	201880	0	2.96E-29
Calophyllum soulattri	R19	P	65	129522	129586	346457	346521	0	2.96E-29
Calophyllum soulattri	R20	F	60	332436	332495	357499	357558	0	3.03E-26
Calophyllum soulattri	R21	F	59	6475	6533	330809	330867	0	1.21E-25
Calophyllum soulattri	R22	F	59	239593	239651	330809	330867	0	1.21E-25
Calophyllum soulattri	R23	P	69	109194	109262	258222	258290	-3	1.63E-25
Calophyllum soulattri	R24	P	62	55790	55851	209746	209807	-1	3.52E-25
Calophyllum soulattri	R25	F	64	131823	131886	209729	209792	-2	2.15E-24
Calophyllum soulattri	R26	P	66	89179	89244	377219	377284	-3	9.13E-24
Calophyllum soulattri	R27	P	59	214776	214834	275833	275891	-1	2.14E-23
Calophyllum soulattri	R28	F	55	319127	319181	354916	354970	0	3.10E-23
Calophyllum soulattri	R29	F	62	28558	28619	122035	122096	-2	3.22E-23
Calophyllum soulattri	R30	P	53	9737	9789	355571	355623	0	4.96E-22
Calophyllum soulattri	R31	P	53	242855	242907	355571	355623	0	4.96E-22
Calophyllum soulattri	R32	F	56	27937	27992	164439	164494	-1	1.30E-21
Calophyllum soulattri	R33	P	56	164388	164443	201076	201131	-1	1.30E-21
Calophyllum soulattri	R34	P	62	26648	26709	129220	129281	-3	1.93E-21
Calophyllum soulattri	R35	F	51	293410	293460	371966	372016	0	7.94E-21
Calophyllum soulattri	R36	P	60	26702	26761	98497	98556	-3	2.80E-20
Calophyllum soulattri	R37	P	60	26702	26761	204977	205036	-3	2.80E-20
Calophyllum soulattri	R38	P	50	27892	27941	201082	201131	0	3.17E-20
Calophyllum soulattri	R39	F	59	23130	23188	121429	121487	-3	1.06E-19
Calophyllum soulattri	R40	F	49	209771	209819	302205	302253	0	1.27E-19
Calophyllum soulattri	R41	P	50	55777	55826	302205	302254	-1	4.76E-18
Calophyllum soulattri	R42	F	50	129228	129277	232526	232575	-1	4.76E-18
Calophyllum soulattri	R43	P	54	214775	214828	249640	249693	-3	8.30E-17
Calophyllum soulattri	R44	P	44	13323	13366	194900	194943	0	1.30E-16
Calophyllum soulattri	R45	P	44	194900	194943	246441	246484	0	1.30E-16
Calophyllum soulattri	R46	F	50	6808	6857	72434	72483	-2	3.50E-16
Calophyllum soulattri	R47	F	50	47455	47504	319675	319724	-2	3.50E-16
Calophyllum soulattri	R48	F	50	72434	72483	239926	239975	-2	3.50E-16
Calophyllum soulattri	R49	F	43	172714	172756	287280	287322	0	5.20E-16
Calophyllum soulattri	R50	F	43	280735	280777	322660	322702	0	5.20E-16
Calophyllum soulattri	R51	F	46	24264	24309	259510	259555	-1	1.12E-15
Calophyllum soulattri	R52	F	46	179014	179059	250296	250341	-1	1.12E-15
Calophyllum soulattri	R53	P	52	26665	26716	129213	129264	-3	1.18E-15
Calophyllum soulattri	R54	P	42	109595	109636	302434	302475	0	2.08E-15
Calophyllum soulattri	R55	P	51	109216	109266	258218	258268	-3	4.46E-15
Calophyllum soulattri	R56	P	48	25177	25224	131844	131891	-2	5.16E-15
Calophyllum soulattri	R57	P	41	23149	23189	200997	201037	0	8.32E-15
Calophyllum soulattri	R58	P	50	26652	26701	232526	232575	-3	1.68E-14
Calophyllum soulattri	R59	P	40	214795	214834	275833	275872	0	3.33E-14
Calophyllum soulattri	R60	F	43	131844	131886	209750	209792	-1	6.71E-14
Calophyllum soulattri	R61	F	39	8852	8890	19476	19514	0	1.33E-13
Calophyllum soulattri	R62	F	39	19476	19514	241970	242008	0	1.33E-13
Calophyllum soulattri	R63	P	39	133606	133644	323120	323158	0	1.33E-13
Calophyllum soulattri	R64	P	39	206848	206886	323347	323385	0	1.33E-13
Calophyllum soulattri	R65	F	42	99676	99717	139468	139509	-1	2.62E-13
Calophyllum soulattri	R66	P	45	25182	25226	209748	209792	-2	2.90E-13
Calophyllum soulattri	R67	P	47	55805	55851	131840	131886	-3	8.89E-13

Calophyllum soulattri	R68	P	41	8530	8570	29022	29062	-1	1.02E-12
Calophyllum soulattri	R69	P	41	8530	8570	105569	105609	-1	1.02E-12
Calophyllum soulattri	R70	P	41	29022	29062	241648	241688	-1	1.02E-12
Calophyllum soulattri	R71	P	41	105569	105609	241648	241688	-1	1.02E-12
Calophyllum soulattri	R72	P	37	55790	55826	302205	302241	0	2.13E-12
Calophyllum soulattri	R73	F	37	146005	146041	146058	146094	0	2.13E-12
Calophyllum soulattri	R74	P	46	50056	50101	221601	221646	-3	3.33E-12
Calophyllum soulattri	R75	F	40	57748	57787	287740	287779	-1	3.99E-12
Calophyllum soulattri	R76	F	40	133565	133604	180834	180873	-1	3.99E-12
Calophyllum soulattri	R77	P	43	26674	26716	129213	129255	-2	4.23E-12
Calophyllum soulattri	R78	P	43	47431	47473	149782	149824	-2	4.23E-12
Calophyllum soulattri	R79	F	36	6822	6857	72448	72483	0	8.52E-12
Calophyllum soulattri	R80	P	36	8210	8245	264558	264593	0	8.52E-12
Calophyllum soulattri	R81	F	36	72448	72483	239940	239975	0	8.52E-12
Calophyllum soulattri	R82	P	36	133610	133645	287739	287774	0	8.52E-12
Calophyllum soulattri	R83	F	36	203459	203494	314398	314433	0	8.52E-12
Calophyllum soulattri	R84	P	36	241328	241363	264558	264593	0	8.52E-12
Calophyllum soulattri	R85	F	45	25182	25226	55805	55849	-3	1.25E-11
Calophyllum soulattri	R86	F	39	8600	8638	214775	214813	-1	1.56E-11
Calophyllum soulattri	R87	P	39	86483	86521	214726	214764	-1	1.56E-11
Calophyllum soulattri	R88	F	39	214775	214813	241718	241756	-1	1.56E-11
Calophyllum soulattri	R89	F	35	47470	47504	319690	319724	0	3.41E-11
Calophyllum soulattri	R90	F	35	109741	109775	262374	262408	0	3.41E-11
Calophyllum soulattri	R91	F	35	287740	287774	323120	323154	0	3.41E-11
Calophyllum soulattri	R92	P	44	8547	8590	29002	29045	-3	4.65E-11
Calophyllum soulattri	R93	P	44	8547	8590	105549	105592	-3	4.65E-11
Calophyllum soulattri	R94	P	44	29002	29045	241665	241708	-3	4.65E-11
Calophyllum soulattri	R95	P	44	89291	89334	377129	377172	-3	4.65E-11
Calophyllum soulattri	R96	P	44	105549	105592	241665	241708	-3	4.65E-11
Calophyllum soulattri	R97	P	38	206890	206927	376083	376120	-1	6.07E-11
Calophyllum soulattri	R98	P	34	20267	20300	209682	209715	0	1.36E-10
Calophyllum soulattri	R99	F	34	57756	57789	87036	87069	0	1.36E-10
Calophyllum soulattri	R100	P	43	26573	26615	208027	208069	-3	1.73E-10
Calophyllum soulattri	R101	P	43	26596	26638	208004	208046	-3	1.73E-10
Calophyllum soulattri	R102	P	43	58896	58938	77560	77602	-3	1.73E-10
Calophyllum soulattri	R103	P	43	182191	182233	364824	364866	-3	1.73E-10
Calophyllum soulattri	R104	P	40	121448	121487	200998	201037	-2	2.34E-10
Calophyllum soulattri	R105	P	37	139572	139608	302899	302935	-1	2.36E-10
Calophyllum soulattri	R106	P	42	8584	8625	275867	275908	-3	6.45E-10
Calophyllum soulattri	R107	P	42	26839	26880	189743	189784	-3	6.45E-10
Calophyllum soulattri	R108	P	42	200248	200289	296712	296753	-3	6.45E-10
Calophyllum soulattri	R109	P	42	241702	241743	275867	275908	-3	6.45E-10
Calophyllum soulattri	R110	P	39	57748	57786	133606	133644	-2	8.88E-10
Calophyllum soulattri	R111	F	39	57748	57786	323120	323158	-2	8.88E-10
Calophyllum soulattri	R112	P	39	78375	78413	377731	377769	-2	8.88E-10
Calophyllum soulattri	R113	F	36	55758	55793	120830	120865	-1	9.20E-10
Calophyllum soulattri	R114	P	36	78152	78187	145733	145768	-1	9.20E-10
Calophyllum soulattri	R115	P	41	8598	8638	249655	249695	-3	2.40E-09
Calophyllum soulattri	R116	P	41	50065	50105	221597	221637	-3	2.40E-09
Calophyllum soulattri	R117	P	41	109538	109578	302483	302523	-3	2.40E-09
Calophyllum soulattri	R118	P	41	241716	241756	249655	249695	-3	2.40E-09
Calophyllum soulattri	R119	F	41	249659	249699	275858	275898	-3	2.40E-09
Calophyllum soulattri	R120	P	38	8601	8638	275854	275891	-2	3.37E-09
Calophyllum soulattri	R121	P	38	241719	241756	275854	275891	-2	3.37E-09
Calophyllum soulattri	R122	F	31	165323	165353	372113	372143	0	8.73E-09
Calophyllum soulattri	R123	F	31	205783	205813	205915	205945	0	8.73E-09
Calophyllum soulattri	R124	P	31	206848	206878	376131	376161	0	8.73E-09
Calophyllum soulattri	R125	F	31	226002	226032	226033	226063	0	8.73E-09
Calophyllum soulattri	R126	F	31	323355	323385	376131	376161	0	8.73E-09
Calophyllum soulattri	R127	P	40	26871	26910	189713	189752	-3	8.88E-09
Calophyllum soulattri	R128	P	37	26665	26701	232526	232562	-2	1.28E-08
Calophyllum soulattri	R129	P	39	129247	129285	279320	279358	-3	3.29E-08
Calophyllum soulattri	R130	F	30	99688	99717	139480	139509	0	3.49E-08
Calophyllum soulattri	R131	F	33	72874	72906	72893	72925	-1	5.40E-08
Calophyllum soulattri	R132	P	38	9086	9123	320256	320293	-3	1.21E-07
Calophyllum soulattri	R133	P	38	242204	242241	320256	320293	-3	1.21E-07
Calophyllum soulattri	R134	P	35	7668	7702	321205	321239	-2	1.83E-07
Calophyllum soulattri	R135	F	35	22675	22709	120826	120860	-2	1.83E-07
Calophyllum soulattri	R136	P	35	25389	25423	208686	208720	-2	1.83E-07
Calophyllum soulattri	R137	F	35	105432	105466	290053	290087	-2	1.83E-07
Calophyllum soulattri	R138	P	35	120826	120860	200186	200220	-2	1.83E-07

Calophyllum soulattri	R139	P	35	240786	240820	321205	321239	-2	1.83E-07
Calophyllum soulattri	R140	F	32	66317	66348	67548	67579	-1	2.09E-07
Calophyllum soulattri	R141	F	32	87036	87067	287748	287779	-1	2.09E-07
Calophyllum soulattri	R142	F	32	198504	198535	209075	209106	-1	2.09E-07
Calophyllum soulattri	R143	P	37	25333	25369	205030	205066	-3	4.47E-07
Calophyllum soulattri	R144	F	37	181260	181296	290644	290680	-3	4.47E-07
Calophyllum soulattri	R145	F	37	280693	280729	322609	322645	-3	4.47E-07
Calophyllum soulattri	R146	F	34	20589	20622	55403	55436	-2	6.88E-07
Calophyllum soulattri	R147	P	34	55818	55851	131840	131873	-2	6.88E-07
Calophyllum soulattri	R148	F	34	120889	120922	149801	149834	-2	6.88E-07
Calophyllum soulattri	R149	F	34	219830	219863	295609	295642	-2	6.88E-07
Calophyllum soulattri	R150	P	31	32217	32247	82262	82292	-1	8.12E-07
Calophyllum soulattri	R151	P	31	110391	110421	293932	293962	-1	8.12E-07
Calophyllum soulattri	R152	F	31	125732	125762	295138	295168	-1	8.12E-07
Calophyllum soulattri	R153	F	31	268042	268072	319312	319342	-1	8.12E-07
Calophyllum soulattri	R154	F	31	325325	325355	325334	325364	-1	8.12E-07
Calophyllum soulattri	R155	P	36	61591	61626	345323	345358	-3	1.64E-06
Calophyllum soulattri	R156	P	36	164514	164549	201552	201587	-3	1.64E-06
Calophyllum soulattri	R157	P	33	25138	25170	302234	302266	-2	2.59E-06
Calophyllum soulattri	R158	F	33	25915	25947	121115	121147	-2	2.59E-06
Calophyllum soulattri	R159	P	33	26729	26761	98497	98529	-2	2.59E-06
Calophyllum soulattri	R160	P	33	26729	26761	204977	205009	-2	2.59E-06
Calophyllum soulattri	R161	F	33	29018	29050	86480	86512	-2	2.59E-06
Calophyllum soulattri	R162	P	33	45237	45269	361093	361125	-2	2.59E-06
Calophyllum soulattri	R163	F	33	86480	86512	105565	105597	-2	2.59E-06
Calophyllum soulattri	R164	P	33	89136	89168	377304	377336	-2	2.59E-06
Calophyllum soulattri	R165	F	33	289274	289306	323128	323160	-2	2.59E-06
Calophyllum soulattri	R166	F	30	8541	8570	214734	214763	-1	3.14E-06
Calophyllum soulattri	R167	P	30	29021	29050	214735	214764	-1	3.14E-06
Calophyllum soulattri	R168	P	30	45164	45193	343170	343199	-1	3.14E-06
Calophyllum soulattri	R169	P	30	105568	105597	214735	214764	-1	3.14E-06
Calophyllum soulattri	R170	F	30	185230	185259	341471	341500	-1	3.14E-06
Calophyllum soulattri	R171	P	30	200968	200997	268166	268195	-1	3.14E-06
Calophyllum soulattri	R172	P	30	206899	206928	376082	376111	-1	3.14E-06
Calophyllum soulattri	R173	F	30	214734	214763	241659	241688	-1	3.14E-06
Calophyllum soulattri	R174	F	30	264336	264365	320582	320611	-1	3.14E-06
Calophyllum soulattri	R175	P	30	275981	276010	322725	322754	-1	3.14E-06
Calophyllum soulattri	R176	P	30	275984	276013	322408	322437	-1	3.14E-06
Calophyllum soulattri	R177	F	35	6345	6379	266854	266888	-3	6.02E-06
Calophyllum soulattri	R178	F	35	239463	239497	266854	266888	-3	6.02E-06
Calophyllum soulattri	R179	P	32	8576	8607	28985	29016	-2	9.74E-06
Calophyllum soulattri	R180	P	32	8576	8607	105532	105563	-2	9.74E-06
Calophyllum soulattri	R181	P	32	28985	29016	241694	241725	-2	9.74E-06
Calophyllum soulattri	R182	R	32	68227	68258	68227	68258	-2	9.74E-06
Calophyllum soulattri	R183	P	32	105532	105563	241694	241725	-2	9.74E-06
Calophyllum soulattri	R184	P	34	13377	13410	194863	194896	-3	2.20E-05
Calophyllum soulattri	R185	F	34	79598	79631	79614	79647	-3	2.20E-05
Calophyllum soulattri	R186	P	34	194863	194896	246495	246528	-3	2.20E-05
Calophyllum soulattri	R187	F	34	198571	198604	284231	284264	-3	2.20E-05
Calophyllum soulattri	R188	F	34	249640	249673	275839	275872	-3	2.20E-05
Calophyllum soulattri	R189	P	31	8401	8431	358300	358330	-2	3.65E-05
Calophyllum soulattri	R190	P	31	8542	8572	86482	86512	-2	3.65E-05
Calophyllum soulattri	R191	P	31	22679	22709	302243	302273	-2	3.65E-05
Calophyllum soulattri	R192	F	31	57756	57786	289274	289304	-2	3.65E-05
Calophyllum soulattri	R193	P	31	86482	86512	241660	241690	-2	3.65E-05
Calophyllum soulattri	R194	P	31	87036	87066	133606	133636	-2	3.65E-05
Calophyllum soulattri	R195	F	31	87036	87066	289274	289304	-2	3.65E-05
Calophyllum soulattri	R196	F	31	87036	87066	323128	323158	-2	3.65E-05
Calophyllum soulattri	R197	P	31	133606	133636	289274	289304	-2	3.65E-05
Calophyllum soulattri	R198	F	31	200186	200216	302243	302273	-2	3.65E-05
Calophyllum soulattri	R199	P	31	241519	241549	358300	358330	-2	3.65E-05
Calophyllum soulattri	R200	F	33	5194	5226	360724	360756	-3	8.03E-05
Calophyllum soulattri	R201	P	33	29464	29496	62242	62274	-3	8.03E-05
Calophyllum soulattri	R202	P	33	89219	89251	377212	377244	-3	8.03E-05
Calophyllum soulattri	R203	P	33	149746	149778	209804	209836	-3	8.03E-05
Calophyllum soulattri	R204	P	33	165529	165561	208839	208871	-3	8.03E-05
Calophyllum soulattri	R205	F	33	238312	238344	360724	360756	-3	8.03E-05
Calophyllum soulattri	R206	F	33	360461	360493	366094	366126	-3	8.03E-05
Calophyllum soulattri	R207	P	33	361441	361473	362339	362371	-3	8.03E-05
Calophyllum soulattri	R208	P	30	9094	9123	320256	320285	-2	1.37E-04
Calophyllum soulattri	R209	F	30	29919	29948	40104	40133	-2	1.37E-04

Calophyllum soulattri	R210	F	30	55747	55776	149733	149762	-2	1.37E-04
Calophyllum soulattri	R211	P	30	56637	56666	56637	56666	-2	1.37E-04
Calophyllum soulattri	R212	P	30	89104	89133	377351	377380	-2	1.37E-04
Calophyllum soulattri	R213	P	30	104598	104627	155119	155148	-2	1.37E-04
Calophyllum soulattri	R214	P	30	116730	116759	314465	314494	-2	1.37E-04
Calophyllum soulattri	R215	F	30	118302	118331	234746	234775	-2	1.37E-04
Calophyllum soulattri	R216	F	30	187988	188017	353001	353030	-2	1.37E-04
Calophyllum soulattri	R217	P	30	214799	214828	249640	249669	-2	1.37E-04
Calophyllum soulattri	R218	P	30	242212	242241	320256	320285	-2	1.37E-04
Calophyllum soulattri	R219	F	32	31762	31793	72563	72594	-3	2.92E-04
Calophyllum soulattri	R220	P	32	187174	187205	268160	268191	-3	2.92E-04
Calophyllum soulattri	R221	P	32	250737	250768	307909	307940	-3	2.92E-04
Calophyllum soulattri	R222	F	32	331378	331409	356320	356351	-3	2.92E-04
Calophyllum soulattri	R223	F	31	6467	6497	22377	22407	-3	1.06E-03
Calophyllum soulattri	R224	F	31	9497	9527	20224	20254	-3	1.06E-03
Calophyllum soulattri	R225	F	31	20224	20254	242615	242645	-3	1.06E-03
Calophyllum soulattri	R226	F	31	22377	22407	239585	239615	-3	1.06E-03
Calophyllum soulattri	R227	F	31	22679	22709	55758	55788	-3	1.06E-03
Calophyllum soulattri	R228	P	31	25408	25438	208671	208701	-3	1.06E-03
Calophyllum soulattri	R229	P	31	38329	38359	323893	323923	-3	1.06E-03
Calophyllum soulattri	R230	P	31	44911	44941	343426	343456	-3	1.06E-03
Calophyllum soulattri	R231	F	31	45609	45639	138318	138348	-3	1.06E-03
Calophyllum soulattri	R232	P	31	55758	55788	200186	200216	-3	1.06E-03
Calophyllum soulattri	R233	F	31	90482	90512	190874	190904	-3	1.06E-03
Calophyllum soulattri	R234	P	31	99710	99740	258051	258081	-3	1.06E-03
Calophyllum soulattri	R235	P	31	117845	117875	363442	363472	-3	1.06E-03
Calophyllum soulattri	R236	P	31	120830	120860	302243	302273	-3	1.06E-03
Calophyllum soulattri	R237	F	31	287748	287778	289274	289304	-3	1.06E-03
Calophyllum soulattri	R238	F	31	322411	322441	322725	322755	-3	1.06E-03
Calophyllum soulattri	R239	F	30	73	102	98	127	-3	3.83E-03
Calophyllum soulattri	R240	F	30	3159	3188	3188	3217	-3	3.83E-03
Calophyllum soulattri	R241	F	30	6438	6467	80515	80544	-3	3.83E-03
Calophyllum soulattri	R242	P	30	7687	7716	290090	290119	-3	3.83E-03
Calophyllum soulattri	R243	F	30	8643	8672	214814	214843	-3	3.83E-03
Calophyllum soulattri	R244	R	30	14269	14298	247385	247414	-3	3.83E-03
Calophyllum soulattri	R245	P	30	26565	26594	208048	208077	-3	3.83E-03
Calophyllum soulattri	R246	P	30	26883	26912	189711	189740	-3	3.83E-03
Calophyllum soulattri	R247	F	30	36596	36625	54497	54526	-3	3.83E-03
Calophyllum soulattri	R248	P	30	61806	61835	345096	345125	-3	3.83E-03
Calophyllum soulattri	R249	P	30	78387	78416	377728	377757	-3	3.83E-03
Calophyllum soulattri	R250	F	30	80515	80544	239556	239585	-3	3.83E-03
Calophyllum soulattri	R251	F	30	99762	99791	206846	206875	-3	3.83E-03
Calophyllum soulattri	R252	F	30	110669	110698	322377	322406	-3	3.83E-03
Calophyllum soulattri	R253	P	30	121463	121492	200993	201022	-3	3.83E-03
Calophyllum soulattri	R254	P	30	142935	142964	222094	222123	-3	3.83E-03
Calophyllum soulattri	R255	P	30	150378	150407	153541	153570	-3	3.83E-03
Calophyllum soulattri	R256	P	30	180603	180632	253279	253308	-3	3.83E-03
Calophyllum soulattri	R257	F	30	213961	213990	307166	307195	-3	3.83E-03
Calophyllum soulattri	R258	F	30	214814	214843	241761	241790	-3	3.83E-03
Calophyllum soulattri	R259	P	30	240805	240834	290090	290119	-3	3.83E-03
Calophyllum soulattri	R260	F	30	248723	248752	356688	356717	-3	3.83E-03
Calophyllum soulattri	R261	F	30	249671	249700	275870	275899	-3	3.83E-03
Calophyllum soulattri	R262	P	30	255414	255443	264187	264216	-3	3.83E-03
Calophyllum soulattri	R263	P	30	266505	266534	323657	323686	-3	3.83E-03
Calophyllum soulattri	R264	F	30	275987	276016	291299	291328	-3	3.83E-03
Calophyllum soulattri	R265	P	30	299361	299390	313142	313171	-3	3.83E-03
Calophyllum soulattri	R266	F	30	348250	348279	348928	348957	-3	3.83E-03

Table S6. The homologous DNA fragment identified among the mitochondrial and chloroplast genomes of *C. soulattri*.

Number	% Identity	Alignment	Mismatches	Gap Openings	Alignment start (chloroplast genome)	Alignment end (chloroplast genome)	Alignment start (mitochondrial genome)	Alignment end (mitochondrial genome)	E-value	Bit Score	MTPT Annotation
1	99.93	2921	1	1	103158	106078	77863	80782	0	5382	*trnV*-GAC
2	99.93	2921	1	1	146904	143984	77863	80782	0	5382	*trnV*-GAC
3	98.22	3036	5	2	46261	43251	1	3012	0	5262	
4	100.00	1452	0	0	21913	23364	3484	4935	0	2682	
5	99.31	1164	2	1	47334	46177	377099	378262	0	2100	
6	99.74	377	1	0	22988	23364	237677	238053	0	691	
7	83.36	565	53	19	46982	47538	89029	89560	6.36E-136	484	
8	73.96	891	174	45	106016	106879	6686	7545	1.48E-82	307	*rrn18*
9	73.96	891	174	45	106016	106879	239804	240663	1.48E-82	307	*rrn18*
10	73.96	891	174	45	144046	143183	239804	240663	1.48E-82	307	*rrn18*
11	73.96	891	174	45	144046	143183	6686	7545	1.48E-82	307	*rrn18*
12	81.19	335	26	16	47728	48050	89552	89861	7.07E-61	235	*trnS*-GGA
13	89.31	187	10	7	33392	33207	249864	250041	4.25E-58	226	*trnD*-GUC
14	81.75	263	37	5	70496	70754	275326	275581	4.28E-53	209	
15	95.65	92	3	1	70308	70399	275179	275269	3.41E-34	147	*trnW*-CCA
16	97.40	77	2	0	114071	113995	290611	290687	9.54E-30	132	*trnN*-GUU
17	96.25	80	3	0	135988	136067	290608	290687	9.54E-30	132	*trnN*-GUU
18	85.12	121	18	0	42406	42286	1633	1753	1.60E-27	124	
19	94.67	75	4	0	55366	55292	85639	85713	2.67E-25	117	*trnM*-CAU
20	100.00	41	0	0	126426	126466	78374	78414	4.53E-13	76.8	
21	80.22	91	14	3	8746	8659	89722	89811	9.82E-10	65.8	
22	100.00	29	0	0	91127	91155	354130	354158	2.12E-06	54.7	
23	96.88	32	1	0	158935	158904	354130	354161	2.12E-06	54.7	

Table S7. RNA editing events identified in the mitochondrial PCGs of *C. soulattri*.

Gene	Base Position	Amino Position	Codon Change	Amino Change	Left Motifs	Edited Site	Right Motifs	Probability
atp1	1039	347	CCC->UCC	Pro->Ser	ATATTCCTACCAATGTGATC	C	CCATTACGGATGGACAAATC	1.00
atp1	1178	393	UCA->UUA	Ser->Leu	GAAACAAGTCTGCGGAAGTT	C	AAAACTAGAATTGGCACAAT	1.00
atp1	1216	406	CUU->UUU	Leu->Phe	AATATCGCGAAGTGGCCGCC	C	TTGCTCAATTTGGATCAGAC	1.00
atp4	38	13	UCU->UUU	Ser->Phe	GTTATTTGCTGCTATTCTAT	C	TATTTGTGCATTAAGTTCGA	0.98
atp4	68	23	UCA->UUA	Ser->Leu	ATTAAGTTCGAAGAAGATCT	C	AATCTATAATGAAGAAATGA	1.00
atp4	97	33	CGU->UGU	Arg->Cys	ATGAAGAAATGATAGTAGCT	C	GTTGTTTTATAGGCTTTATC	0.97
atp4	194	65	UCG->UUG	Ser->Leu	CCAGGCTATTCAGGAAGAAT	C	GCAGCAATTCCTCAATCCTA	1.00
atp4	227	76	CCU->CUU	Pro->Leu	CAATCCTAACGAAGTAGTTC	C	TCCGGAATCCAATGAACAAC	1.00
atp4	229	77	CCG->UUG	Pro->Leu	ATCCTAACGAAGTAGTTCCT	C	CGGAATCCAATGAACAACAA	0.90
atp4	230	77	CCG->UUG	Pro->Leu	TCCTAACGAAGTAGTTCCTC	C	GGAATCCAATGAACAACAAC	1.00
atp4	374	125	UCA->UUA	Ser->Leu	CCGAAACCTAAATGTTAAGT	C	AGCAACACTTCCAAATGCCA	1.00
atp4	386	129	CCA->CUA	Pro->Leu	TGTTAAGTCAGCAACACTTC	C	AAATGCCACTTCTTCCCGTC	0.99
atp4	395	132	ACU->AUU	Thr->Ile	AGCAACACTTCCAAATGCCA	C	TTCTTCCCGTCGCATCCGTC	0.99
atp6	37	13	CCA->UCA	Pro->Ser	TGTATTTCTCATTCACAAAT	C	CATCTTTGTTTATGCTGCTA	1.00
atp6	79	27	CUG->UUG	Leu->Leu	CTCTCAGTTTAGTCTTACTT	C	TGGTTCATTTGGTTACTAAA	0.97
atp6	173	58	CCG->CUG	Pro->Leu	TTATGATTTCGTGCTGAACC	C	GGTAAACGAACAAATAGGTG	1.00
atp6	229	77	CGC->UGC	Arg->Cys	TTAAACAAAAGTTTTTCCCT	C	GCATCTTGGTCACTTTTACT	1.00
atp6	254	85	UCG->UUG	Ser->Leu	CTTGGTCACTTTTACTTTTT	C	GTTATTTCGTAATCTCCAGG	1.00
atp6	262	88	CGU->UGU	Arg->Cys	CTTTTACTTTTTCGTTATTT	C	GTAATCTCCAGGGTATGATA	1.00
atp6	270	90	CUC->CUU	Leu->Leu	TTTTCGTTATTTCGTAATCT	C	CAGGGTATGATACCTTATAG	0.97
atp8	47	16	UCA->UUA	Ser->Leu	TTTTACACAATTCTTCTGGT	C	ATGCCTTTTTTTCTTTATTT	1.00
ccmB	28	10	CAU->UAU	His->Tyr	GACTCTTTCTTGAACTATAT	C	ATAAACAGATCTTCTCCTCC	0.99
ccmB	71	24	UCA->UUA	Ser->Leu	ACCAATCACGAGTTTTTCTT	C	ATTCCTCTCGTATATCATCA	1.00
ccmB	80	27	UCG->UUG	Ser->Leu	GAGTTTTTCTTCATTCCTCT	C	GTATATCATCATAACGCCCT	0.97
ccmB	87	29	AUC->AUU	Ile->Ile	TCTTCATTCCTCTCGTATAT	C	ATCATAACGCCCTTAATGCT	0.99
ccmB	128	43	UCA->UUA	Ser->Leu	AGGTTTTGAAAAAGACTTTT	C	ATGTCATTCCCATTTAGGTC	1.00
ccmB	137	46	UCC->UUC	Ser->Phe	AAAAGACTTTTCATGTCATT	C	CCATTTAGGTCTGATTCGGA	1.00
ccmB	148	50	CUG->UUG	Leu->Leu	CATGTCATTCCCATTTAGGT	C	TGATTCGGATCCCTCCGTTG	0.93
ccmB	154	52	CGG->UGG	Arg->Trp	ATTCCCATTTAGGTCTGATT	C	GGATCCCTCCGTTGTTTCCT	0.99
ccmB	160	54	CCU->UCU	Pro->Ser	ATTTAGGTCTGATTCGGATC	C	CTCCGTTGTTTCCTTTTCCT	0.97
ccmB	164	55	CCG->CUG	Pro->Leu	AGGTCTGATTCGGATCCCTC	C	GTTGTTTCCTTTTCCTCCCG	0.96
ccmB	172	58	CCU->UCU	Pro->Ser	TTCGGATCCCTCCGTTGTTT	C	CTTTTCCTCCCGCACCTTTT	0.99
ccmB	179	60	CCU->CUU	Pro->Leu	CCCTCCGTTGTTTCCTTTTC	C	TCCCGCACCTTTTCCTCGAA	0.99
ccmB	181	61	CCC->UCC	Pro->Ser	CTCCGTTGTTTCCTTTTCCT	C	CCGCACCTTTTCCTCGAAAT	1.00
ccmB	193	65	CCU->UUU	Pro->Phe	CTTTTCCTCCCGCACCTTTT	C	CTCGAAATGAGAAAGAAGAT	0.95
ccmB	194	65	CCU->UUU	Pro->Phe	TTTTCCTCCCGCACCTTTTC	C	TCGAAATGAGAAAGAAGATG	1.00
ccmB	313	105	CGU->UGU	Arg->Cys	TTCAAATAAGTTGTGTTTTC	C	GTGGTTTTCCCATCTTACAA	1.00
ccmB	337	113	CCG->UUG	Pro->Leu	GTTTTCCCATCTTACAACTT	C	CGTACCAATTCGGTAGATCC	0.96
ccmB	338	113	CCG->UUG	Pro->Leu	TTTTCCCATCTTACAACTTC	C	GTACCAATTCGGTAGATCCG	0.98
ccmB	380	127	UCA->UUA	Ser->Leu	AATGGATTGGTTAAACATTT	C	ATTAGGGAGCCTGGTCTTGA	1.00
ccmB	424	142	CGU->UGU	Arg->Cys	TTCTGTGTGGTATTCATTCT	C	GTTCGGCTCTTGGAATCACA	0.99
ccmB	428	143	UCG->UUG	Ser->Leu	GTGTGGTATTCATTCTCGTT	C	GGCTCTTGGAATCACATCCA	1.00
ccmB	467	156	UCG->UUG	Ser->Leu	CAGCAGTGGTTGGAACAGCT	C	GCAAAATCCAACCACTTCAC	0.98

ccmB	475	159	CCA->UUA	Pro->Leu	GTTGGAACAGCTCGCAAAAT	C	CAACCACTTCACCTACTTTA	1.00
ccmB	476	159	CCA->UUA	Pro->Leu	TTGGAACAGCTCGCAAAATC	C	AACCACTTCACCTACTTTAT	1.00
ccmB	485	162	UCA->UUA	Ser->Leu	CTCGCAAAATCCAACCACTT	C	ACCTACTTTATTGCCCCCAA	0.99
ccmB	503	168	CCA->CUA	Pro->Leu	TTCACCTACTTTATTGCCCC	C	AACCCTTTCTCGTATCTCTA	0.98
ccmB	512	171	UCU->UUU	Ser->Phe	TTTATTGCCCCCAACCCTTT	C	TCGTATCTCTATTGAAACAG	0.99
ccmB	551	184	UCA->UUA	Ser->Leu	AGAATGGTTTCATGTTCTTT	C	ATCGATTGGTTATTCCTCTC	1.00
ccmB	554	185	UCG->UUG	Ser->Leu	ATGGTTTCATGTTCTTTCAT	C	GATTGGTTATTCCTCTCCGT	0.99
ccmB	566	189	UCC->UUC	Ser->Phe	TCTTTCATCGATTGGTTATT	C	CTCTCCGTTCGTATCTCTTT	1.00
ccmB	569	190	UCU->UUU	Ser->Phe	TTCATCGATTGGTTATTCCT	C	TCCGTTCGTATCTCTTTTTC	0.98
ccmB	572	191	CCG->CUG	Pro->Leu	ATCGATTGGTTATTCCTCTC	C	GTTCGTATCTCTTTTTCCAA	1.00
ccmB	596	199	UCG->UUG	Ser->Leu	CGTATCTCTTTTTCCAATTT	C	GGTCTCGATTAGTTTACAAG	1.00
ccmC	5	2	UCC->UUC	Ser->Phe	AGGACCAAATTCTCACATGT	C	CGTTTCGTTATTACAACCTT	1.00
ccmC	76	26	CGG->UGG	Arg->Trp	CGCAAATTATCATTGGGTCT	C	GGTTGTTCTTAACAGCGATG	0.98
ccmC	103	35	CAU->UAU	His->Tyr	TCTTAACAGCGATGGCTATT	C	ATTTAAGTTTTCGGGTAGCA	1.00
ccmC	115	39	CGG->UGG	Arg->Trp	TGGCTATTCATTTAAGTTTT	C	GGGTAGCACCACTAGATCTT	0.99
ccmC	133	45	CUU->UUU	Leu->Phe	TTCGGGTAGCACCACTAGAT	C	TTCAACAAGGTGGAAATTCT	0.99
ccmC	179	60	GCG->GUG	Ala->Val	TCTGTATGTACATGTTCCTG	C	GGCTCGGATGAGTATTCTTC	1.00
ccmC	184	62	CGG->UGG	Arg->Trp	ATGTACATGTTCCTGCGGCT	C	GGATGAGTATTCTTCTTTAT	0.98
ccmC	331	111	CGG->UGG	Arg->Trp	CCTTAGTGACTGGGGGGTTT	C	GGGGAAGACCTATGTGGGGC	1.00
ccmC	395	132	UCG->UUG	Ser->Leu	TTTAACCTCTGTATTCATCT	C	GTTCCTTATTTACCTGGGTG	1.00
ccmC	399	133	UUC->UUU	Phe->Phe	ACCTCTGTATTCATCTCGTT	C	CTTATTTACCTGGGTGCACT	0.99
ccmC	400	134	CUU->UUU	Leu->Phe	CCTCTGTATTCATCTCGTTC	C	TTATTTACCTGGGTGCACTG	1.00
ccmC	436	146	CCU->UCU	Pro->Ser	CACTGTGTTTTCAAAAGCTT	C	CTGTCGAACCGGCTTCTATT	1.00
ccmC	446	149	CCG->CUG	Pro->Leu	TCAAAAGCTTCCTGTCGAAC	C	GGCTTCTATTTCAATCCGTG	1.00
ccmC	458	153	UCA->UUA	Ser->Leu	TGTCGAACCGGCTTCTATTT	C	AATCCGTGCTGGACCGATCG	0.98
ccmC	463	155	CGU->UGU	Arg->Cys	AACCGGCTTCTATTTCAATC	C	GTGCTGGACCGATCGATATA	1.00
ccmC	467	156	GCU->GUU	Ala->Val	GGCTTCTATTTCAATCCGTG	C	TGGACCGATCGATATACCAA	1.00
ccmC	473	158	CCG->CUG	Pro->Leu	TATTTCAATCCGTGCTGGAC	C	GATCGATATACCAATAATCA	1.00
ccmC	521	174	UCG->UUG	Ser->Leu	AGTCAACTGGTGGAATACAT	C	GCATCAACCTGGGAGCATTA	1.00
ccmC	548	183	UCU->UUU	Ser->Phe	ACCTGGGAGCATTAGTCGAT	C	TGGTACATCAATACATGTTC	0.97
ccmC	568	190	CCU->UCU	Pro->Ser	CTGGTACATCAATACATGTT	C	CTATGCCCATTCCAATCTTG	1.00
ccmC	575	192	CCC->CUC	Pro->Leu	ATCAATACATGTTCCTATGC	C	CATTCCAATCTTGTCTAACT	1.00
ccmC	605	202	UCC->UUC	Ser->Phe	CTTGTCTAACTTTGCTAACT	C	CCCCTTCTCAACCCCTATCT	1.00
ccmC	608	203	CCC->CUC	Pro->Leu	GTCTAACTTTGCTAACTCCC	C	CTTCTCAACCCCTATCTTCT	0.99
ccmC	614	205	UCA->UUA	Ser->Leu	CTTTGCTAACTCCCCCTTCT	C	AACCCCTATCTTCTTTGTTC	1.00
ccmC	650	217	CCU->CUU	Pro->Leu	TGTTCTGGAAACACGTCTTC	C	TATTCCATCTTTTCTCGAAT	1.00
ccmC	656	219	CCA->CUA	Pro->Leu	GGAAACACGTCTTCCTATTC	C	ATCTTTTCTCGAATCTCCTT	1.00
ccmC	673	225	CCU->UCU	Pro->Ser	TTCCATCTTTTCTCGAATCT	C	CTTTCACGGAAGAAATAGAA	0.99
ccmFc	39	13	UUC->UUU	Phe->Phe	TTTTTCTTTTTTATTACTTT	C	ATGGTCGTGCCTCGTGGCAC	0.90
ccmFc	50	17	CCU->CUU	Pro->Leu	TATTACTTTCATGGTCGTGC	C	TCGTGGCACGGCAGCACCCG	1.00
ccmFc	52	18	CGU->UGU	Arg->Cys	TTACTTTCATGGTCGTGCCT	C	GTGGCACGGCAGCACCCGTA	0.98
ccmFc	103	35	CCC->UCC	Pro->Ser	GGTTTCTCAGTAGAGATGTT	C	CCATAGGTGCCCCTTCTTCC	0.97
ccmFc	122	41	UCC->UUC	Ser->Phe	TCCCATAGGTGCCCCTTCTT	C	CAATGGTACTATAATTCCTA	1.00
ccmFc	146	49	CCU->CUU	Pro->Leu	TGGTACTATAATTCCTATTC	C	TATCTCTTCATTCCCTCTTT	0.99
ccmFc	155	52	UCA->UUA	Ser->Leu	AATTCCTATTCCTATCTCTT	C	ATTCCCTCTTTTGGTCTATC	0.99
ccmFc	310	104	CGU->UGU	Arg->Cys	GAGTTAGAAACGTCTTATTT	C	GTTTCGTTCCCGTTCTTCAT	0.94

ccmFc	412	138	CGU->UGU	Arg->Cys	TGCTCTGTTTACTATTCTTT	C	GTACTTTCTTCTCTTTATCA	1.00
ccmFc	752	251	UCU->UUU	Ser->Phe	ACATAGAGGAGTGTGCATCT	C	TATGTTGGGTGTTCTTCTGT	1.00
ccmFc	884	295	UCU->UUU	Ser->Phe	TCATTTACATGGACCCACTT	C	TCATTCCATTTGTGGGAATT	1.00
ccmFc	1166	389	UCG->UUG	Ser->Leu	ATTTACGGATCTATATGCTT	C	GATTGGAACTGGAAGTTCCA	1.00
ccmFc	1240	414	CGG->UGG	Arg->Trp	TGCCTTTTATTTTTTTTATT	C	GGAAAGGATTTATGTTGGCT	1.00
ccmFc	1274	425	UCG->UUG	Ser->Leu	GTTGGCTTCGTTAGGAGGCT	C	GCGTAGTTTGTTACGTCAGC	0.98
ccmFc	1321	441	CGA->UGA	Arg->End	AGGATAAGTTGCGTTGGCAT	C	GAGAAAGTTCCGTGGAGTTC	0.96
ccmFn	38	13	CCG->CUG	Pro->Leu	GTTTCATTATTCGTTATTTC	C	GGGTCTTTTCGTTGCATTCA	0.99
ccmFn	98	33	CCC->CUC	Pro->Leu	ACCAGCGTTTGGTGCAGCAC	C	CGCATTTTGGTGTATTCTTC	1.00
ccmFn	137	46	UCG->UUG	Ser->Leu	TCTTTCTTTCCTTGGTCTTT	C	GTTCCGTCATATTCCTAATA	0.98
ccmFn	142	48	CGU->UGU	Arg->Cys	CTTTCCTTGGTCTTTCGTTC	C	GTCATATTCCTAATAACTTA	0.99
ccmFn	151	51	CCU->UCU	Pro->Ser	GTCTTTCGTTCCGTCATATT	C	CTAATAACTTATCCAATTAC	0.99
ccmFn	248	83	UCA->UUA	Ser->Leu	TCATGAGGGTAGTATTTTAT	C	ATGGTGTCGGATCCCAAGTT	1.00
ccmFn	256	86	CGG->UGG	Arg->Trp	GTAGTATTTTATCATGGTGT	C	GGATCCCAAGTTTTTATGGA	1.00
ccmFn	263	88	CCA->CUA	Pro->Leu	TTTATCATGGTGTCGGATCC	C	AAGTTTTTATGGATTCCTTC	1.00
ccmFn	283	95	CUU->UUU	Leu->Phe	CAAGTTTTTATGGATTCCTT	C	TTTGTTACCGGGGTCGACCC	1.00
ccmFn	365	122	UCG->UUG	Ser->Leu	TCTTTTTTATTCCTTTGTCT	C	GAACTTCGTGAAGAACTCCA	0.97
ccmFn	372	124	UUC->UUU	Phe->Phe	TATTCCTTTGTCTCGAACTT	C	GTGAAGAACTCCATTCTATC	0.99
ccmFn	716	239	UCA->UUA	Ser->Leu	GTTTTTCTCTCTTTTTTTAT	C	AGCGAGTTCTGATCCTTTTG	0.91
ccmFn	754	252	CGU->UGU	Arg->Cys	TTGTTCGAAATTTCTTCGTT	C	GTACCGAACCGCTTGCAGAA	1.00
ccmFn	776	259	UCA->UUA	Ser->Leu	TACCGAACCGCTTGCAGAAT	C	AAATCCTGTTCTACAAGATC	0.99
ccmFn	803	268	UCA->UUA	Ser->Leu	TGTTCTACAAGATCCTATAT	C	AGCTATACATCCTCCTTGCA	0.97
ccmFn	1298	433	CCA->CUA	Pro->Leu	TTTAACCGTAGGCATCTTGC	C	AGGAAGTTGGTGGGCTCATC	1.00
ccmFn	1315	439	CAU->UAU	His->Tyr	TGCCAGGAAGTTGGTGGGCT	C	ATCATGAATTAGGTCGGGGT	1.00
ccmFn	1330	444	CGG->UGG	Arg->Trp	GGGCTCATCATGAATTAGGT	C	GGGGTGGCTGGTGGTTTTGG	1.00
ccmFn	1381	461	CGG->UGG	Arg->Trp	AAAATGCTTCTTTTATGCCT	C	GGGTATTAGCCACAGCTCGT	1.00
ccmFn	1399	467	CGU->UGU	Arg->Cys	CTCGGGTATTAGCCACAGCT	C	GTATTCATTCAGTAATTCTA	1.00
ccmFn	1442	481	UCG->UUG	Ser->Leu	CCTTCTTCATTCTTGGACCT	C	GTTTCTTAATATTGTTACTT	1.00
ccmFn	1466	489	CCA->CUA	Pro->Leu	TCTTAATATTGTTACTTTTC	C	ATGCTGTGTCTCAGGAACCT	1.00
ccmFn	1478	493	UCA->UUA	Ser->Leu	TACTTTTCCATGCTGTGTCT	C	AGGAACCTTTTCAATACGGT	1.00
cox1	15	5	GUC->GUU	Val->Val	TTTCTTATGACAAATCTGGT	C	CGATGGCTCTTTTCCACTAA	1.00
cox1	1149	383	CUC->CUU	Leu->Leu	GCACATTTCCATTATGTACT	C	TCTATGGGAGCCGTTTTTGC	0.92
cox2	33	11	AUC->AUU	Ile->Ile	TTTCTAGACTTCCTCACAAT	C	TCTCCTTGTGATGCAGCGGA	0.96
cox2	71	24	UCU->UUU	Ser->Phe	GGAACCATGGCAATTAGGAT	C	TCAAGATGCAGCAACACCTA	1.00
cox2	253	85	CGG->UGG	Arg->Trp	GAACTACTATCGAGATTCTT	C	GGACCATCTTTCCTAGTATC	0.99
cox2	278	93	CCU->CUU	Pro->Leu	CATCTTTCCTAGTATCATCC	C	TATGTTCATTGCTATACCAT	0.99
cox2	379	127	CGG->UGG	Arg->Trp	CTATTGGACATCAATGGTAT	C	GGACTTATGAGTATTCAGAC	1.00
cox2	443	148	ACG->AUG	Thr->Met	ACTCACTTTTGACAGTTATA	C	GATTCCAGAAGATGATCTAG	1.00
cox2	476	159	UCA->UUA	Ser->Leu	TGATCTAGAATTGGGTCAAT	C	ACGTTTATTAGAAGTGGACA	1.00
cox2	557	186	CCU->CUU	Pro->Leu	TGTAACATCTGCTGATGTAC	C	TCATAGTTGGGCTGTACCTT	1.00
cox2	632	211	UCG->UUG	Ser->Leu	TTTAAATCAGATCTCTATTT	C	GGTACAACGAGAAGGAGTTT	1.00
cox3	245	82	CCU->CUU	Pro->Leu	CAAAGTCGTACAATTAGGAC	C	TCGATATGGTTTTATTCTGT	1.00
cox3	311	104	UCU->UUU	Ser->Phe	TTTTGCTTTTTTTTGGGCTT	C	TTCTCATTCTTCTTTGGCAC	1.00
cox3	314	105	UCU->UUU	Ser->Phe	TGCTTTTTTTTGGGCTTCTT	C	TCATTCTTCTTTGGCACCTA	1.00
cox3	413	138	CCC->CUC	Pro->Leu	AATCCCTTTTCTTAATACCC	C	CATTCTCCTTTCATCCGGAG	0.99
matR	236	79	UCC->UUC	Ser->Phe	CGATCCCAAGTTTTTTTACT	C	CATTCAGAAAGTCTTTTCCG	0.92

matR	326	109	CCA->CUA	Pro->Leu	TGTACTACTATCGGCCCTAC	C	AGGCAACATCTACCTACACA	1.00
matR	413	138	UCG->UUG	Ser->Leu	GATTGTTGAGAGAATCAGAT	C	GGTTCTATTAAGGACAGGTC	0.99
matR	1676	559	UCC->UUC	Ser->Phe	CGGAGACATCGTAAATTGGT	C	CGCGGGCATCGCGATAAGTC	1.00
matR	1717	573	CGC->UGC	Arg->Cys	TTCTGTCCTACTACAGGTGC	C	GCGACAACTTTTACCAAGTC	1.00
matR	1731	577	UAC->UAU	Tyr->Tyr	AGGTGCCGCGACAACTTTTA	C	CAAGTCCGAACGATTGTCGA	0.99
matR	1753	585	CAC->UAC	His->Tyr	AAGTCCGAACGATTGTCGAC	C	ACCAGATCCGCTGGTCTGCA	1.00
matR	1823	608	CCA->CUA	Pro->Leu	CTCGGCGCGGAATATAATCC	C	AAAGTACTCCAAAGACTCAA	1.00
matR	1841	614	UCA->UUA	Ser->Leu	CCCAAAGTACTCCAAAGACT	C	AAATATAGTAAATAAAGAAG	1.00
mttB	22	8	CCC->UUC	Pro->Phe	TGGTTTACGTGTTACTGGTT	C	CCGGAAGAGTTAATATCTCC	0.95
mttB	23	8	CCC->UUC	Pro->Phe	GGTTTACGTGTTACTGGTTC	C	CGGAAGAGTTAATATCTCCA	1.00
mttB	39	13	AUC->AUU	Ile->Ile	GTTCCCGGAAGAGTTAATAT	C	TCCATTAGCAAAACCCTTTC	0.99
mttB	42	14	UCC->UCU	Ser->Ser	CCCGGAAGAGTTAATATCTC	C	ATTAGCAAAACCCTTTCTTA	0.95
mttB	99	33	AUC->AUU	Ile->Ile	GTATTTTGTTTGTACACAAT	C	AACGGAGGCCTCCTCGACAT	1.00
mttB	111	37	CUC->CUU	Leu->Leu	TACACAATCAACGGAGGCCT	C	CTCGACATATATTGCAACGT	0.99
mttB	173	58	UCA->UUA	Ser->Leu	TCATCTTTCCTTTAATAAGT	C	ATCAAATTTGGTGCTTTTTG	0.99
mttB	239	80	CCU->CUU	Pro->Leu	GGACGAAATACAATCGATTC	C	TCCATTTAAGTGGTTCTCGC	0.98
mttB	242	81	CCA->CUA	Pro->Leu	CGAAATACAATCGATTCCTC	C	ATTTAAGTGGTTCTCGCTTC	0.98
mttB	255	85	UUC->UUU	Phe->Phe	ATTCCTCCATTTAAGTGGTT	C	TCGCTTCTCCTTGTTCCTGT	0.93
mttB	257	86	UCG->UUG	Ser->Leu	TCCTCCATTTAAGTGGTTCT	C	GCTTCTCCTTGTTCCTGTTC	1.00
mttB	284	95	UCC->UUU	Ser->Phe	CCTTGTTCCTGTTCCTAACT	C	CTCCCCGGGTAGTTCCCAAT	0.95
mttB	285	95	UCC->UUU	Ser->Phe	CTTGTTCCTGTTCCTAACTC	C	TCCCCGGGTAGTTCCCAATG	1.00
mttB	287	96	UCC->UUC	Ser->Phe	TGTTCCTGTTCCTAACTCCT	C	CCCGGGTAGTTCCCAATGTT	0.99
mttB	290	97	CCG->CUG	Pro->Leu	TCCTGTTCCTAACTCCTCCC	C	GGGTAGTTCCCAATGTTTGG	1.00
mttB	318	106	UUC->UUU	Phe->Phe	TCCCAATGTTTGGCACTTTT	C	ATACTTCGTGGGTGCAACAT	1.00
mttB	348	116	UUC->UUU	Phe->Phe	GGGTGCAACATCAACAAATT	C	GCTTATGATCAAGTTACAAC	0.90
nad1	2	1	ACG->AUG	Thr->Met	AGTGAATAGAAAATCGAAAA	C	GTACATAGCTGTTCCAGCCG	0.98
nad1	167	56	UCG->UUG	Ser->Leu	ACAACCTCTAGCAGATGGTT	C	GAAATTGATTCTAAAAGAAC	1.00
nad1	215	72	UCC->UUC	Ser->Phe	ACCAAGTAGTGCTAATTTCT	C	CCTTTTTAGAATGGCTCCAG	1.00
nad1	265	89	CGG->UGG	Arg->Trp	TTATGTTAAGTCTGGTCGCT	C	GGGCCGTTGTACCTTTTGAT	1.00
nad1	307	103	CCG->UUG	Pro->Leu	ATGGTATGGTATTGTCAGAT	C	CGAACATAGGTCTACTTTAT	0.99
nad1	308	103	CCG->UUG	Pro->Leu	TGGTATGGTATTGTCAGATC	C	GAACATAGGTCTACTTTATT	1.00
nad1	490	164	CCC->UCU	Pro->Ser	CTGTACTAATATGTGTAGGT	C	CCCGTAATTCGAGTGAGATT	1.00
nad1	492	164	CCC->UCU	Pro->Ser	GTACTAATATGTGTAGGTCC	C	CGTAATTCGAGTGAGATTGT	0.99
nad1	493	165	CGU->UGU	Arg->Cys	TACTAATATGTGTAGGTCCC	C	GTAATTCGAGTGAGATTGTC	1.00
nad1	500	167	UCG->UUG	Ser->Leu	ATGTGTAGGTCCCCGTAATT	C	GAGTGAGATTGTCATGGCGC	1.00
nad1	536	179	UCU->UUU	Ser->Phe	GGCGCAAAAGCGGATATGGT	C	TGGTATTCCCTTGTTCCCTG	0.98
nad1	571	191	CUC->UUU	Leu->Phe	TCCCTGTATTGGTTATGTTC	C	TCATTTCTTGTCTAGCAGAA	1.00
nad1	573	191	CUC->UUU	Leu->Phe	CCTGTATTGGTTATGTTCCT	C	ATTTCTTGTCTAGCAGAAAC	1.00
nad1	635	212	UCA->UUA	Ser->Leu	CCCAGAAGCGGAAGCTGAAT	C	AGTTGCAGGCTATAATGTAG	1.00
nad1	725	242	CCA->CUA	Pro->Leu	TATGATCTTAATGAGTGGTC	C	ATGCACATCGCTCTTTCCAG	1.00
nad1	734	245	UCG->UUG	Ser->Leu	AATGAGTGGTCCATGCACAT	C	GCTCTTTCCAGGAGGTTGGC	1.00
nad1	743	248	CCA->CUA	Pro->Leu	TCCATGCACATCGCTCTTTC	C	AGGAGGTTGGCCGCCTATCC	1.00
nad1	755	252	CCG->CUG	Pro->Leu	GCTCTTTCCAGGAGGTTGGC	C	GCCTATCCTAGATCTTCCCA	1.00
nad1	898	300	CGG->UGG	Arg->Trp	ATCAATTAATGGGACTTGGC	C	GGAAAGTGTTCTTGCCCCTA	1.00
nad1	928	310	CGG->UGG	Arg->Trp	TCTTGCCCCTATCATTAGCT	C	GGGTAGTCCCCGTTTCTGGT	1.00
nad1	937	313	CCC->UCC	Pro->Ser	TATCATTAGCTCGGGTAGTC	C	CCGTTTCTGGTGTTTTAGTC	0.99

nad2	26	9	UCC->UUC	Ser->Phe	CAATCTTTTTTTAGCGGTTT	C	CCCAGAGATCTTTATCATTA	1.00
nad2	56	19	UCC->UUC	Ser->Phe	CTTTATCATTAATGCAACCT	C	CATTTTGCTCATTCATGGAG	1.00
nad2	223	75	CUU->UUU	Leu->Phe	CCCATTTATTCTGGAATAAT	C	TTTTTAGGAGGGACAATTTT	1.00
nad2	308	103	UCU->UUU	Ser->Phe	CATTTCGATGTGTTTCGATT	C	TTCCGAACAAGAGAGGTTTG	1.00
nad2	311	104	UCC->UUC	Ser->Phe	TTCGATGTGTTTCGATTCTT	C	CGAACAAGAGAGGTTTGATG	1.00
nad2	367	123	CGC->UGC	Arg->Cys	TATTAATTCTACTTTCTACT	C	GCAGTATGCTCTTTATGATC	1.00
nad2	394	132	CAU->UAU	His->Tyr	TGCTCTTTATGATCTCGGCT	C	ATGATTCAATTGCCATGTAT	1.00
nad2	401	134	UCA->UUA	Ser->Leu	TATGATCTCGGCTCATGATT	C	AATTGCCATGTATTTAGCTA	1.00
nad2	428	143	CCU->CUU	Pro->Leu	CATGTATTTAGCTATTGAGC	C	TCAAAGTTTATGTTTTTATG	1.00
nad2	497	166	UCG->UUG	Ser->Leu	ATTTTCCACGGAAGCCGGCT	C	GAAATATTTGATCTTAGGTG	1.00
nad2	523	175	CCC->UCC	Pro->Ser	ATTTGATCTTAGGTGCATTT	C	CCTCTGGAATCTTATTGTTT	1.00
nad2	788	263	UCU->UUU	Ser->Phe	TGCGCCTAAAATTTCTATTT	C	TGCTAATATTTCACGTGTTT	1.00
nad2	800	267	UCA->UUA	Ser->Leu	TTCTATTTCTGCTAATATTT	C	ACGTGTTTCTATTTATGGTT	0.98
nad2	809	270	UCU->UUU	Ser->Phe	TGCTAATATTTCACGTGTTT	C	TATTTATGGTTCCTATGGAG	1.00
nad2	920	307	CCU->CUU	Pro->Leu	CCAAACGAAAGTAAAAAGAC	C	TCTAGCTCATAGTTCAATTG	1.00
nad2	928	310	CAU->UAU	His->Tyr	AAGTAAAAAGACCTCTAGCT	C	ATAGTTCAATTGGACATGTA	0.99
nad2	958	320	CGU->UGU	Arg->Cys	TTGGACATGTAGGTTATATT	C	GTACTGGTTTCTCATGTGGA	1.00
nad2	962	321	ACU->AUU	Thr->Ile	ACATGTAGGTTATATTCGTA	C	TGGTTTCTCATGTGGAACCA	1.00
nad2	1127	376	UCG->UUG	Ser->Leu	AGCCAAAACGAATCCTATTT	C	GGCTATTACCTTCTCCATTA	1.00
nad2	1246	416	CCA->UCA	Pro->Ser	GTGGGGCTTACTTCCTAGCC	C	CAGTGGGAGTAGTGACTAGC	1.00
nad2	1276	426	CGU->UGU	Arg->Cys	TAGTGACTAGCGTTATAGGT	C	GTTTTTATTATATACGCTTA	1.00
nad2	1298	433	GCG->GUG	Ala->Val	TTTTTATTATATACGCTTAG	C	GAAAAGAATGTTTTTTGATA	1.00
nad2	1400	467	UCA->UUA	Ser->Leu	GACTTCCTCTTTCATTACTT	C	ATCCTTTCTATATCCCTCTC	0.99
nad2	1403	468	UCC->UUC	Ser->Phe	TTCCTCTTTCATTACTTCAT	C	CTTTCTATATCCCTCTCCCT	0.99
nad2	1408	470	CUA->UUA	Leu->Leu	CTTTCATTACTTCATCCTTT	C	TATATCCCTCTCCCTTGTTC	0.98
nad2	1416	472	CCC->CCU	Pro->Pro	ACTTCATCCTTTCTATATCC	C	TCTCCCTTGTTCTCAGTTAC	0.98
nad2	1457	486	UCA->UUA	Ser->Leu	TCATCAAATGGCACTCAGTT	C	ATATCTTTAAGTTCGAGCAG	0.99
nad3	5	2	UCA->UUA	Ser->Leu	CAAGTGTTCTGTGGTGATGT	C	AGAATTTGGACCTATTTGTA	0.96
nad3	44	15	CCG->CUG	Pro->Leu	TATCTATTTAGTGATCAGTC	C	GATAGTCTCTTTGATCCCAC	1.00
nad3	62	21	CCA->CUA	Pro->Leu	TCCGATAGTCTCTTTGATCC	C	ACTCGGTCTTCCTTTTCTAT	0.99
nad3	146	49	UCC->UUC	Ser->Phe	CGAATGTGGTTTCGATCCTT	C	CGGTGATGCCAGAAGTCGTT	1.00
nad3	208	70	CUU->UUU	Leu->Phe	TTTCAATTTTATTTATTATT	C	TTGATCTGGAAGTCACCTTT	1.00
nad3	317	106	UCU->UUU	Ser->Phe	ATTGATTTTAACGATTGGAT	C	TCTGTATGAATGGAAAAGGG	1.00
nad3	349	117	CGG->UGG	Arg->Trp	GGAAAAGGGGTGCTTTGGAT	C	GGGAGTAATCACTAATGATA	1.00
nad4	29	10	UCU->UUU	Ser->Phe	ACATTTCTGTGAATGCTATT	C	TGATTTAAGTGGTCTTATTC	1.00
nad4	74	25	ACU->AUU	Thr->Ile	TCCCGTGCTAGGAAGCATTA	C	TCCTCTTTTCATTCCAAATT	1.00
nad4	77	26	CCU->CUU	Pro->Leu	CGTGCTAGGAAGCATTACTC	C	TCTTTTCATTCCAAATTCAA	1.00
nad4	107	36	CCG->CUG	Pro->Leu	TCCAAATTCAAGAATACGGC	C	GATACGATTGATTGGTCTAT	0.98
nad4	158	53	CCU->CUU	Pro->Leu	TATTACTTTTTTGTATTCCC	C	TGTTCCTCGGATACAATTCG	1.00
nad4	164	55	CCU->CUU	Pro->Leu	TTTTTTGTATTCCCCTGTTC	C	TCGGATACAATTCGATCCTT	0.99
nad4	166	56	CGG->UGG	Arg->Trp	TTTTGTATTCCCCTGTTCCT	C	GGATACAATTCGATCCTTCT	1.00
nad4	197	66	UCU->UUU	Ser->Phe	CGATCCTTCTACGGCCAAAT	C	TCAATTTGTAGAAAGCCTTC	0.99
nad4	362	121	ACA->AUA	Thr->Ile	TTATGGGAAAGAGTATATTA	C	AGCATCTCTAATTCGTGAAT	1.00
nad4	368	123	UCU->UUU	Ser->Phe	GAAAGAGTATATTACAGCAT	C	TCTAATTCGTGAATTTCTAA	1.00
nad4	376	126	CGU->UGU	Arg->Cys	ATATTACAGCATCTCTAATT	C	GTGAATTTCTAATGATCGCC	1.00
nad4	403	135	CGC->UGC	Arg->Cys	TTCTAATGATCGCCGTGTTC	C	GCATGCTGGATCTTCTACTA	1.00

nad4	433	145	CUU->UUU	Leu->Phe	ATCTTCTACTATTCTATGTT	C	TTCCCGAAAGCGTCTTAATC	1.00
nad4	436	146	CCC->UUC	Pro->Phe	TTCTACTATTCTATGTTCTT	C	CCGAAAGCGTCTTAATCCCT	0.97
nad4	437	146	CCC->UUC	Pro->Phe	TCTACTATTCTATGTTCTTC	C	CGAAAGCGTCTTAATCCCTA	0.98
nad4	608	203	UCA->UUA	Ser->Leu	AACCACCGATTTACAAATAT	C	ATTAACCACAGAATTTAGTG	1.00
nad4	659	220	UCU->UUU	Ser->Phe	AATCTTTCTATGGATTGCTT	C	TTTCGCCTCTTTCGCCGTCA	1.00
nad4	767	256	CCU->CUU	Pro->Leu	CGTCATCTTGGCAGGAATTC	C	TTTAAAATTGGGAACCTACG	1.00
nad4	836	279	UCC->UUC	Ser->Phe	TCCAGAAGCGACACTTTCTT	C	CACTCCTTTCATTTATACTC	0.99
nad4	856	286	CCA->UUA	Pro->Leu	CCACTCCTTTCATTTATACT	C	CAAGCGCGATTGCTATAATA	0.98
nad4	857	286	CCA->UUA	Pro->Leu	CACTCCTTTCATTTATACTC	C	AAGCGCGATTGCTATAATAT	1.00
nad4	896	299	UCA->UUA	Ser->Leu	ATATACTTCCTTGACCACTT	C	AAGACAGATCGATCTTAAGA	1.00
nad4	977	326	CCG->CUG	Pro->Leu	GACTATTGGTATGTTTAGTC	C	GAACATACAGGGAATTGGAG	1.00
nad4	1006	336	CUA->UUA	Leu->Leu	AGGGAATTGGAGGTAGCATT	C	TACCGATGTTAAGTCATGGA	0.95
nad4	1010	337	CCG->CUG	Pro->Leu	AATTGGAGGTAGCATTCTAC	C	GATGTTAAGTCATGGACTGG	1.00
nad4	1101	367	UAC->UAU	Tyr->Tyr	ACTCGACTTGTTAGATATTA	C	GGAGGTTTAGTGAGCACCAT	0.96
nad4	1172	391	UCA->UUA	Ser->Leu	TACTTTGGCCAATATGAGTT	C	ACCTGGTACTAGCAGCTTTA	1.00
nad4	1205	402	CCC->CUU	Pro->Leu	CAGCTTTATCGGGGAATTTC	C	CATCTCAGTAGGAGCTTTCC	1.00
nad4	1206	402	CCC->CUU	Pro->Leu	AGCTTTATCGGGGAATTTCC	C	ATCTCAGTAGGAGCTTTCCA	0.96
nad4	1211	404	UCA->UUA	Ser->Leu	TATCGGGGAATTTCCCATCT	C	AGTAGGAGCTTTCCAAAGAA	1.00
nad4	1355	452	UCA->UUA	Ser->Leu	CCTCCATAAATTCTCCGATT	C	AAATGGCAGAGAAGTTTCCA	0.99
nad4	1373	458	UCC->UUC	Ser->Phe	TTCAAATGGCAGAGAAGTTT	C	CATATTTATACCTTTTCTTG	1.00
nad4	1405	469	CGG->UGG	Arg->Trp	CTTTTCTTGTTGGAGTTGTT	C	GGATGGGTGTTCACCCCAAA	1.00
nad4	1417	473	CAC->UAC	His->Tyr	GAGTTGTTCGGATGGGTGTT	C	ACCCCAAAGTGTTCCCGGAC	1.00
nad4	1433	478	CCG->CUG	Pro->Leu	TGTTCACCCCAAAGTGTTCC	C	GGACTGCATGCATACATCCG	1.00
nad4L	55	19	CGG->UGG	Arg->Trp	TTATCTTTATTTTAGGTATT	C	GGGGAATCCTCCTTAATAGA	1.00
nad4L	86	29	CCU->CUU	Pro->Leu	CCTTAATAGACGAAATATTC	C	TATTATGTCAATGCCAATTG	0.98
nad4L	95	32	UCA->UUA	Ser->Leu	ACGAAATATTCCTATTATGT	C	AATGCCAATTGAATCAATGT	1.00
nad4L	100	34	CCA->UCA	Pro->Ser	ATATTCCTATTATGTCAATG	C	CAATTGAATCAATGTTATTA	1.00
nad4L	110	37	UCA->UUA	Ser->Leu	TATGTCAATGCCAATTGAAT	C	AATGTTATTAGCTGTGAATT	1.00
nad4L	158	53	UCG->UUG	Ser->Leu	TTTGGTATTTTCCGTTTCTT	C	GGATGATATGATGGGTCAAT	1.00
nad4L	179	60	UCA->UUA	Ser->Leu	GGATGATATGATGGGTCAAT	C	ATTTGCTTCATTGGTTTCAA	1.00
nad4L	188	63	UCA->UUA	Ser->Leu	GATGGGTCAATCATTTGCTT	C	ATTGGTTTCAACGGTGGCAG	1.00
nad4L	197	66	UCA->UUA	Ser->Leu	ATCATTTGCTTCATTGGTTT	C	AACGGTGGCAGCTGCGGAAT	1.00
nad5	155	52	CCG->CUG	Pro->Leu	TGCTTTTTATGAAGTCGCAC	C	GGGAGCTAGTGCTTGCTATC	1.00
nad5	374	125	CCA->CUA	Pro->Leu	CATTTTTACTTTTTTTATGC	C	AATGTTGGTGACTGGAGATA	1.00
nad5	398	133	UCU->UUU	Ser->Phe	GTTGGTGACTGGAGATAACT	C	TCTTCAATTATTCCTGGGAT	1.00
nad5	539	180	CCU->CUU	Pro->Leu	AGGTGATTTTGGATTAGCTC	C	TGGGATTTCGGGCCGTTTTA	0.98
nad5	548	183	UCG->UUG	Ser->Leu	TGGATTAGCTCCTGGGATTT	C	GGGCCGTTTTACTCTCTTTC	1.00
nad5	553	185	CGU->UGU	Arg->Cys	TAGCTCCTGGGATTTCGGGC	C	GTTTTACTCTCTTTCAAACA	0.99
nad5	629	210	UCU->UUU	Ser->Phe	CCCCAGAAATTCTTGGATTT	C	TTGCAATATGAGATTGAATG	1.00
nad5	676	226	CUU->UUU	Leu->Phe	CTCTTATTTGTATTTTACTT	C	TTATTGGTGCTGTTGGGAAA	1.00
nad5	713	238	UCG->UUG	Ser->Leu	GAAATCCGCACAGATAGGAT	C	GCATACTTGGTCACCCGATG	1.00
nad5	725	242	UCA->UUA	Ser->Leu	GATAGGATCGCATACTTGGT	C	ACCCGATGCTATGGAGGGTC	1.00
nad5	764	255	UCG->UUG	Ser->Leu	TCCCACTCCAGTATCTGCTT	C	GATTCATGCAGCTACTATGG	1.00
nad5	1310	437	UCA->UUA	Ser->Leu	TTCTTATTACTCTTTTCGTT	C	ACTTTTTCTAACATTTCTAG	1.00
nad5	1490	497	CCC->CUC	Pro->Leu	CAATTTTTGGGCCAATTCCC	C	CTTTGTACTACCAAAAAATG	1.00
nad5	1550	517	ACC->AUC	Thr->Ile	GTTTGCTGCTCCAACCATTA	C	CAAACTAATACCTATTCTGT	1.00

nad5	1580	527	UCA->UUA	Ser->Leu	ACCTATTCTGTTTAGTACTT	C	AGGTGCTTCTGTTGCGTATA	1.00
nad5	1589	530	UCU->UUU	Ser->Phe	GTTTAGTACTTCAGGTGCTT	C	TGTTGCGTATAATGTAAATC	0.99
nad5	1610	537	CCC->CUC	Pro->Leu	TGTTGCGTATAATGTAAATC	C	CGTAGCGGATCAATTCCAAC	1.00
nad5	1895	632	UCA->UUA	Ser->Leu	CTTTGCAATGTTACTTGGTT	C	AACTCTATTTGTGACCTTTT	1.00
nad5	1958	653	UCA->UUA	Ser->Leu	TTCTTGGGTAGATAATCGAT	C	ATCTTTCATTTTGATAGTGA	0.99
nad6	26	9	CCC->CUC	Pro->Leu	ACTTTCTGTTTTGTCGAGCC	C	CGCTTTGGTCTCTGGTTTGA	1.00
nad6	53	18	GCA->GUA	Ala->Val	GGTCTCTGGTTTGATGGTTG	C	ACGTGCTAAAAATCCGGTAC	1.00
nad6	89	30	UCC->UUC	Ser->Phe	GGTACATTCCGTTTTGTTTT	C	CATCCTAGTTTTTCGCGATA	1.00
nad6	103	35	CGC->UGC	Arg->Cys	TGTTTTCCATCCTAGTTTTT	C	GCGATACTTCAGGGTTACTT	0.93
nad6	146	49	UCC->UUC	Ser->Phe	TTTGTTAGGTCTCGACTTCT	C	CGCTATGATCTTCTCAGTAG	1.00
nad6	161	54	UCA->UUA	Ser->Leu	CTTCTCCGCTATGATCTTCT	C	AGTAGTTCATATAGGAGCTA	1.00
nad6	169	57	CAU->UAU	His->Tyr	CTATGATCTTCTCAGTAGTT	C	ATATAGGAGCTATAGCCGTT	1.00
nad6	463	155	CCU->UCU	Pro->Ser	ATTTCGTCTGGTTTTTGCTT	C	CTAGTCTTATTTTATTAGTA	0.96
nad7	38	13	UCG->UUG	Ser->Leu	GCAAATCAAAAATTTCACTT	C	GAATTTCGGACCTCAACATC	1.00
nad7	45	15	UUC->UUU	Phe->Phe	AAAAATTTCACTTCGAATTT	C	GGACCTCAACATCCTGCTGC	1.00
nad7	77	26	UCA->UUA	Ser->Leu	TCCTGCTGCTCATGGTGTTT	C	ACGATCAGTATTGGAAATGA	0.99
nad7	83	28	UCA->UUA	Ser->Leu	TGCTCATGGTGTTTCACGAT	C	AGTATTGGAAATGAACGGAG	1.00
nad7	200	67	UCU->UUU	Ser->Phe	TCTTCAAGCTTTACCTTATT	C	TGATCGTTTAGACTATGTTT	1.00
nad7	316	106	CGU->UGU	Arg->Cys	AATATATACGAGTCTTATTC	C	GTGAAATAACTCGAATTTCA	1.00
nad7	335	112	UCA->UUA	Ser->Leu	CCGTGAAATAACTCGAATTT	C	AAATCATTCACTTGCTTTAA	1.00
nad7	344	115	UCA->UUA	Ser->Leu	AACTCGAATTTCAAATCATT	C	ACTTGCTTTAACTACTCATG	1.00
nad7	383	128	UCA->UUA	Ser->Leu	TGCTATGGATGTGGGAGCAT	C	AACTCCGTTCCTGTGGGCTT	1.00
nad7	578	193	UCA->UUA	Ser->Leu	CGACGAATTAGAAGAGATGT	C	AACCGGCAACCGTATCTGGA	1.00
nad7	679	227	CCA->UCA	Pro->Ser	GTGGTGTAATGTTAAGAGGT	C	CAGGGGTATGCTGGGATTTG	1.00
nad7	724	242	CAU->UAU	His->Tyr	AAGCAGCACCTTACGATGTT	C	ATGACCAATTGGATCCTGAC	1.00
nad7	739	247	CCU->UUU	Pro->Phe	ATGTTCATGACCAATTGGAT	C	CTGACGTACCAGTAGGTACC	1.00
nad7	740	247	CCU->UUU	Pro->Phe	TGTTCATGACCAATTGGATC	C	TGACGTACCAGTAGGTACCA	1.00
nad7	769	257	CGC->UGC	Arg->Cys	CAGTAGGTACCAGAGGAGAT	C	GCTATGATCGTTACTGTATC	1.00
nad7	789	263	AUC->AUU	Ile->Ile	CGCTATGATCGTTACTGTAT	C	CGTATCGAAGAGATGCGACA	0.92
nad7	926	309	UCA->UUA	Ser->Leu	GAAACTATCCATGGAATCCT	C	AATTCACCATTTCGAACTTT	1.00
nad7	963	321	UCC->UCU	Ser->Ser	CTTTATACAGAAGGTTTTTC	C	GTACCAGCTTCTTCTACCTA	0.94
nad7	1057	353	CGU->UGU	Arg->Cys	GAAGCAATCGCCCCTACCGT	C	GTAAAATAAGAGCACCTGGC	0.99
nad7	1088	363	UCA->UUA	Ser->Leu	AGCACCTGGCTTTGCCCATT	C	ACAAGGACTCGATTCTATGT	1.00
nad7	1103	368	UCU->UUU	Ser->Phe	CCATTCACAAGGACTCGATT	C	TATGTCCAAACATCACATGC	1.00
nad7	1124	375	CCA->CUA	Pro->Leu	TATGTCCAAACATCACATGC	C	AGCAGATGTGGTCACCATCA	1.00
nad7	1137	379	GUC->GUU	Val->Val	CACATGCCAGCAGATGTGGT	C	ACCATCATAGGTACTCAAGA	0.94
nad9	92	31	UCU->UUU	Ser->Phe	ATCGGAACATGGGAATAGAT	C	TGATACCAATTCAGATTACC	1.00
nad9	167	56	UCG->UUG	Ser->Leu	TTATACAAGGGTTCAAGTTT	C	GATCGATATTTGCGGAGTTG	1.00
nad9	190	64	CAU->UAU	His->Tyr	TCGATATTTGCGGAGTTGAT	C	ATCCCTCTCGAAAACGAAGA	1.00
nad9	298	100	CCG->UCG	Pro->Ser	ACGAAGTAACACGAATATCT	C	CGGTAGTCAGTCTATTTCCA	1.00
nad9	328	110	CGG->UGG	Arg->Trp	GTCTATTTCCATCAGCCGGC	C	GGTGGGAGCGAGAAGTTTGG	1.00
nad9	368	123	UCC->UUC	Ser->Phe	GGATATGTTTGGTGTTTCTT	C	CATCAATCATCCGGATCTAC	1.00
nad9	398	133	UCA->UUA	Ser->Leu	TCCGGATCTACGCCGTATAT	C	AACAGATTATGGTTTCGAGG	1.00
nad9	439	147	CUU->UUU	Leu->Phe	GTCATCCATTACGAAAAGAC	C	TTCCTCTGAGTGGATATGTT	0.98
rpl10	308	103	UCA->UUA	Ser->Leu	CATGGACCAAGATCTGCTTT	C	ATTATATGGGCAATACCGAT	0.98
rpl5	35	12	UCA->UUA	Ser->Leu	TTTTCATTATGAAGATGTAT	C	ACGTCAGGATCCCTTGCTCA	0.94

rpl5	47	16	CCC->CUC	Pro->Leu	AGATGTATCACGTCAGGATC	C	CTTGCTCAAAGTGAATCACG	0.97
rpl5	844	282	CGC->UGC	Arg->Cys	TGATTAAAAAAATCCCTCCT	C	GCGGTTTAATCTATTTCTTG	0.92
rps12	104	35	CCG->CUG	Pro->Leu	CCAGAAGCAAGGAGTATGCC	C	GCGTCTTTCAACGAGAACAC	1.00
rps12	196	66	CAC->UAC	His->Tyr	ATCGACATGATATATTTGCT	C	ACATTCCGGGTGAGGGTCAT	1.00
rps3	64	22	CGG->UGG	Arg->Trp	ATCGTAGTTCAGATTCAAGT	C	GGTTCAGTGATTATTATTAT	1.00
rps3	69	23	UUC->UUU	Phe->Phe	AGTTCAGATTCAAGTCGGTT	C	AGTGATTATTATTATGGTTT	0.99
rps3	92	31	UCA->UUA	Ser->Leu	TGATTATTATTATGGTTTTT	C	AGTGTATCAAGATGTCAATC	0.99
rps3	371	124	UCA->UUA	Ser->Leu	AAAGAAGAATCTTTCTAAAT	C	AGTTCGGGTCAGCGGGGCCT	0.96
rps3	572	191	UCG->UUG	Ser->Leu	GCGCTTCTCCTTGAATTTTT	C	GGTCATGCAATATTTTTTGA	0.99
rps3	749	250	UCG->UUG	Ser->Leu	CGCTTTTTTTGTAGAAAGCT	C	GACCAGCGAGAAAAAGTGTT	0.97
rps3	896	299	CCA->CUA	Pro->Leu	CGGTGCTACCTTCTTCTTTC	C	AAGGGTTGGGGTGTATAATA	0.95
rps3	1214	405	CCG->CUG	Pro->Leu	TGTTTATCAAAGTGCTTCCC	C	GATTGCTCAAGACATCTCTT	1.00
rps3	1352	451	UCA->UUA	Ser->Leu	TATATGTTGCTCAGGTCGAT	C	AAAAGGCGCAAAAATAGCTA	1.00
rps3	1405	469	CGU->UGU	Arg->Cys	GAAACTATGGAAAAACATCT	C	GTAATGTATTTAACCAGAAA	0.98
rps3	1438	480	CCU->UCU	Pro->Ser	ACCAGAAAATCGATTATGCT	C	CTGCGGAAGTATCTACTCGT	0.99
rps3	1469	490	UCA->UUA	Ser->Leu	ATCTACTCGTTACGGAATCT	C	AGGTGTCAAAGTGTGGATCT	1.00
rps4	155	52	UCA->UUA	Ser->Leu	TCTTAACAGTTACATCCAAT	C	ACAAACTACACGAAAGTTGC	1.00
rps4	175	59	CCC->UCC	Pro->Ser	CACAAACTACACGAAAGTTG	C	CCCTTTTTTATGGAGATTTA	1.00
rps4	257	86	CCA->CUA	Pro->Leu	TATCCCTTTTCTACTCAATC	C	AGAAACAAGATCGGACGTTA	0.99
rps4	269	90	UCG->UUG	Ser->Leu	ACTCAATCCAGAAACAAGAT	C	GGACGTTATTCTGGTTCGTC	1.00
rps4	326	109	CCG->CUG	Pro->Leu	TATTCCTCAAGCAAGGCAGC	C	GATAAGTCATCGAAGGGTTT	1.00
rps4	920	307	UCG->UUG	Ser->Leu	CGAACTACCTACTCATTTTT	C	GGAGGTGAATCATAGAACAC	0.96
rps4	931	311	CAU->UAU	His->Tyr	CTCATTTTTCGGAGGTGAAT	C	ATAGAACACTAAAAGCTGTG	0.95
rps4	956	319	UCU->UUU	Ser->Phe	AACACTAAAAGCTGTGGTAT	C	TTATGGACCTAACATAGGTC	1.00
rps4	1021	341	CGG->UGG	Arg->Trp	AAGATTTCAACCTTCTTCTT	C	GGAGCGGAAATGGACGTGGA	0.98
sdh4	122	41	ACC->AUC	Thr->Ile	GAGCTCGAAGACAAAGAGAA	C	CGGGCTTTTCCAAAGAATTA	0.95

Table S8. Ka/Ks ratios of PCGs in the mitochondrial genome of *C. soulattri.*

**Species vs Species**	***atp1***	***atp4***	***atp6***	***atp8***	***atp9***	***ccmB***	***ccmC***	***ccmFc***	***ccmFn***	***cob***	***cox1***	***cox2***	***cox3***	***matR***	***mttB***	***nad1***	***nad2***	***nad3***	***nad4***	***nad4L***	***nad5***	***nad6***	***nad7***	***nad9***	***rpl5***	***rpl10***	***rps3***	***rps4***	***rps12***
C. soullatri vs P. edulis	0.272	1.169	0.284	0.463	0.022	1.324	0.722	0.590	0.657	0.300	0.168	0.318	0.120	0.522	0.212	0.820	0.274	0.313	0.544	0.133	0.211	0.795	0.228	0.286	0.565	0.551	0.594	0.714	0.203
